# Toll-like receptor-4 null mutation causes fetal loss and fetal growth restriction associated with impaired maternal immune tolerance in mice

**DOI:** 10.1038/s41598-021-95213-1

**Published:** 2021-08-16

**Authors:** Hon Y. Chan, Lachlan M. Moldenhauer, Holly M. Groome, John E. Schjenken, Sarah A. Robertson

**Affiliations:** 1 Robinson Research Institute and Adelaide Medical School, University of Adelaide, Adelaide, SA 5005 Australia; 2 The Priority Research Centre for Reproductive Science and School of Environmental and Life Sciences, University of Newcastle, Callaghan, NSW 2308 Australia

**Keywords:** Reproductive disorders, Infertility, Developmental biology, Intrauterine growth, Immunology, Mucosal immunology

## Abstract

Maternal immune adaptation to accommodate pregnancy depends on sufficient availability of regulatory T (Treg) cells to enable embryo implantation. Toll-like receptor 4 is implicated as a key upstream driver of a controlled inflammatory response, elicited by signals in male partner seminal fluid, to initiate expansion of the maternal Treg cell pool after mating. Here, we report that mice with null mutation in *Tlr4 *(*Tlr4*^*−/−*^) exhibit impaired reproductive outcomes after allogeneic mating, with reduced pregnancy rate, elevated mid-gestation fetal loss, and fetal growth restriction, compared to *Tlr4*^+/+^ wild-type controls. To investigate the effects of TLR4 deficiency on early events of maternal immune adaptation, TLR4-regulated cytokines and immune regulatory microRNAs were measured in the uterus at 8 h post-mating by qPCR, and Treg cells in uterus-draining lymph nodes were evaluated by flow cytometry on day 3.5 post-coitum. *Ptgs2* encoding prostaglandin-endoperoxide synthase 2, cytokines *Csf2*, *Il6*, *Lif,* and *Tnf*, chemokines *Ccl2*, *Cxcl1*, *Cxcl2*, and *Cxcl10*, and microRNAs *miR-155*, *miR-146a*, and *miR-223* were induced by mating in wild-type mice, but not, or to a lesser extent, in *Tlr4*^*−/−*^ mice. CD4^+^ T cells were expanded after mating in *Tlr4*^+/+^ but not *Tlr4*^−/−^ mice, with failure to expand peripheral CD25^+^FOXP3^+^ NRP1^*−*^ or thymic CD25^+^FOXP3^+^ NRP1^+^ Treg cell populations, and fewer Treg cells expressed Ki67 proliferation marker and suppressive function marker CTLA4. We conclude that TLR4 is an essential mediator of the inflammation-like response in the pre-implantation uterus that induces generation of Treg cells to support robust pregnancy tolerance and ensure optimal fetal growth and survival.

## Introduction

At the outset of pregnancy the female immune response must sense and adapt to paternally-derived fetal alloantigens by generating regulatory T (Treg) cells required to facilitate and promote implantation^1,2^. This sensitisation occurs in the female reproductive tract prior to embryo implantation as a consequence of a controlled local inflammatory response that is elicited at mating by seminal fluid from the male partner^3,4^. The female response is characterised by transient induction in the uterus of a wide array of cytokines and chemokines including *Csf2*, *Csf3, Il6, Cxcl1, Il1a* and *Lif*^5^, as well as *miR-155* and other microRNAs (miRNAs)^6,7^*.* The surge in cytokines and miRNAs causes leukocytes—including neutrophils, macrophages, and dendritic cells (DCs)—to influx into the uterine mucosa^8–10^. Recruited antigen presenting cells then take up seminal fluid antigens, traffic to draining lymph nodes, and stimulate both thymic-derived and peripheral Treg cells to proliferate^11,12^. In turn, the expanded populations of Treg cells circulate via the peripheral blood and are recruited into the uterus where they assist embryo implantation, by constraining inflammation, suppressing generation of anti-fetal effector responses, and supporting the process of uterine vascular remodelling required for robust placental development^1,2,13^. Insufficient numbers or impaired function of Treg cells results in implantation failure, fetal loss, and/or fetal growth restriction (FGR) in mice^14–16^, and is implicated as a common cause of infertility and adverse pregnancy outcomes in women^2,17^.

Some of the key signaling agents in seminal fluid have been identified, including TGFB, PGE2, and CD38 in seminal plasma^4^. These factors have pro-tolerogenic properties and drive the adaptive immune response to generate immune-regulatory Treg cells from naïve T cells^18–20^. Recently, we identified Toll-like receptor 4 (TLR4) as an additional key player mediating the female immune response to seminal fluid^21^. In microarray experiments to evaluate gene pathways regulated by seminal fluid contact^21^, TLR4, its adaptor molecule MYD88, and the TLR4 ligand lipopolysaccharide (LPS), were each predicted to be major upstream regulators of the uterine genes induced by seminal fluid contact. In particular, TLR4 is required to mediate interaction between sperm-bound signals and uterine epithelial cells, which causes transcriptional induction of neutrophil-regulating cytokines IL6, CSF3, and CXCL2^22^.

While TLR4 signaling is commonly associated with activation of innate immunity, recent studies have demonstrated that TLR4 also modulates T cell phenotypes in the adaptive immune response, as a result of both direct effects in T cells, and indirect regulation of antigen presenting cell phenotypes and function. TLR4 signaling induces cytokines and chemokines that regulate dendritic cell and macrophage dynamics, and influence their phenotype to impact the strength and balance of the resulting T cell response^23–25^. Other cytokines induced by TLR4 control neutrophils^26,27^, that in turn can influence Treg cell generation and phenotype^28^. These consequences of TLR4 ligation suggest that maternal TLR4 might be a key regulator for generation of Treg cells at the outset of pregnancy.

Our studies to date indicate that maternal TLR4 activation is an important driver of the female response to seminal fluid, but have not reported on the consequences of perturbing TLR4 signaling for Treg cell activation and pregnancy outcome. In this study, we utilized mice with a null mutation in the *Tlr4* gene to test the hypotheses that maternal TLR4 is required for induction of maternal immune tolerance, and that disruption of TLR4-mediated seminal fluid signaling impacts establishment of healthy pregnancy. Our results indicate that maternal TLR4 is essential for optimal fetal growth and pregnancy outcome, and implicate a mechanism involving TLR4-mediated induction of cytokines and expansion of Treg cells at the outset of pregnancy.

## Results

### Effect of maternal TLR4 on d 17.5 pc fetal outcomes

Initially, we evaluated the physiological requirement for TLR4 in pregnancy by investigating late gestation pregnancy outcomes in *Tlr4*^*−/−*^ mice and *Tlr4*^+*/*+^ BALB/c wild-type control mice, after mating with intact C57Bl/6 (B6) males to generate allogeneic pregnancies with maternal, but not fetal, TLR4 deficiency. Mice were killed on d 17.5 pc, and the uterus was excised to enable counting of the number and viability of implantation sites, and weighing of individual dissected fetal and placental tissues.

**Figure 1:**
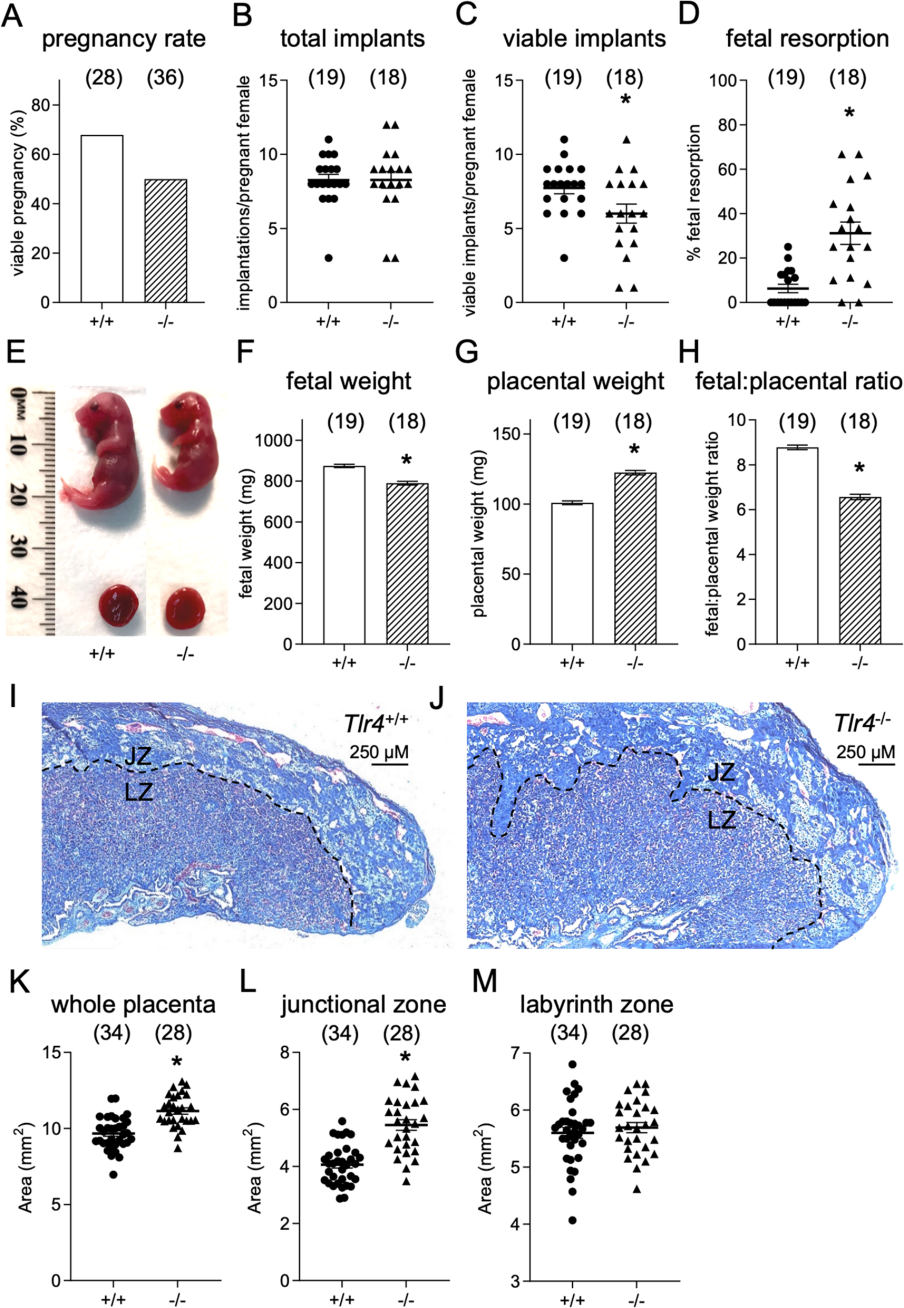
Effect of maternal TLR4 deficiency on pregnancy outcome parameters. Pregnancy outcomes were assessed on d 17.5 pc from *Tlr4*^+*/*+^ (+/+) or *Tlr4*^*−/−*^ (−/−) females mated with B6 males. (**A**) Percentage of matings that led to pregnancy, (**B**) number of total implantation sites per viable pregnancy, (**C**) number of viable implantations per viable pregnancy, (**D**) resorption rate per viable pregnancy, (**E**) representative image of a fetus and placenta from +/+ or −/− pregnancies, (**F**) fetal weight, (**G**) placental weight, (**H**) fetal to placental weight ratio. Symbols depict individual mice (n = 18–19/group) or individual placentae (n = 14–17 dams with viable pregnancy/group, with 2 placentas from each dam) and data are shown as mean ± SEM. Number of mice per group are shown in parentheses. Placentas were collected on d 17.5 pc from *Tlr4*^+*/*+^ **(I)** and *Tlr4*^*−/−*^ (**J**) dams and stained with Masson’s trichrome, to allow quantification of (**K**) total placental area, (**L**) junctional zone area, and (**M**) labyrinth zone area. *JZ *junctional zone, *LZ* labyrinth zone. For (**A**), data are compared by Chi-square test (no significant difference). For (**B**)–(**D**) and (**K**)–(**M**), data are compared by unpaired Sidak *t* test or Mann–Whitney test depending on normality of data distribution. For (**F**)–(**H**), data are estimated marginal mean ± SEM analysed using Mixed Model Linear Repeated Measure ANOVA, with mother as subject. *p < 0.05.

A smaller proportion of *Tlr4*^*−/−*^ females progressed from mating to viable pregnancy (18/36 in *Tlr4*^*−*/*−*^, versus 19/28 in *Tlr4*^+/+^), but the difference was not significant by Chi-square test (Fig. 1A). Within viable pregnancies, there was no difference in the number of total implantation sites (Fig. 1B), but *Tlr4*^*−/−*^ females had a 23% decrease in viable fetuses compared to *Tlr4*^+*/*+^ females (*p* = 0.027, Fig. 1C), associated with a striking fivefold increase in the proportion of resorbing fetuses (*p* < 0.0001, Fig. 1D). Most resorptions were small (< 3 mm) hemorrhagic masses indicating mid-gestation demise. Live fetuses of *Tlr4*^*−/−*^ females were visibly smaller (Fig. 1E), and weighed 9.7% less than fetuses of *Tlr4*^+*/*+^ females (*p* < 0.0001, Fig. 1F). Placental weight was increased by 20% in *Tlr4*^*−*/*−*^ mice (*p* < 0.0001, Fig. 1G), resulting in a 25% reduction in fetal-to-placental weight ratio, a measure of placental transport efficiency, in *Tlr4*^*−/−*^ compared to *Tlr4*^+*/*+^ females (*p* < 0.0001, Fig. 1H). Histochemical analysis of mid-saggital placental sections (Fig. 1I,J) showed that greater placental weight was largely due to an expanded placental junctional zone region in *Tlr4*^*−*/*−*^ compared to *Tlr4*^+/+^ dams (Fig. 1K,L), as opposed to the placental labyrinth which was not proportionately increased (Fig. 1M). These data show that maternal TLR4 deficiency adversely impacts pregnancy outcome, associated with high rates of fetal loss and extensive fetal growth restriction (FGR).

### Effect of maternal TLR4 deficiency on cytokine expression in the uterus

The pregnancy phenotype in *Tlr4*^*−/−*^ mice is consistent with impaired implantation, potentially associated with a disrupted maternal uterine immune response^13–16^. Maternal immune adaption in early pregnancy commences when signals from seminal fluid interact with the uterine endometrium after mating to induce an array of cytokines and chemokines in a controlled inflammatory response^21^. Previously, we showed TLR4 is required for seminal fluid-induced expression of uterine cytokines, with reduced production of *Csf3, Cxcl2, Cxcl10, Il6, Lif,* and *Tnf* in *Tlr4*^*−/−*^ females after mating^21^. To expand insight on uterine genes induced by TLR4 signaling that are specifically implicated in the adaptive immune response, several additional cytokines, chemokines and immune regulatory genes were assessed in new endometrial samples from *Tlr4*^+*/*+^ and *Tlr4*^*−/−*^ females at 8 h pc after mating with B6 males, as well as estrous control females, by qPCR analysis.

**Figure 2:**
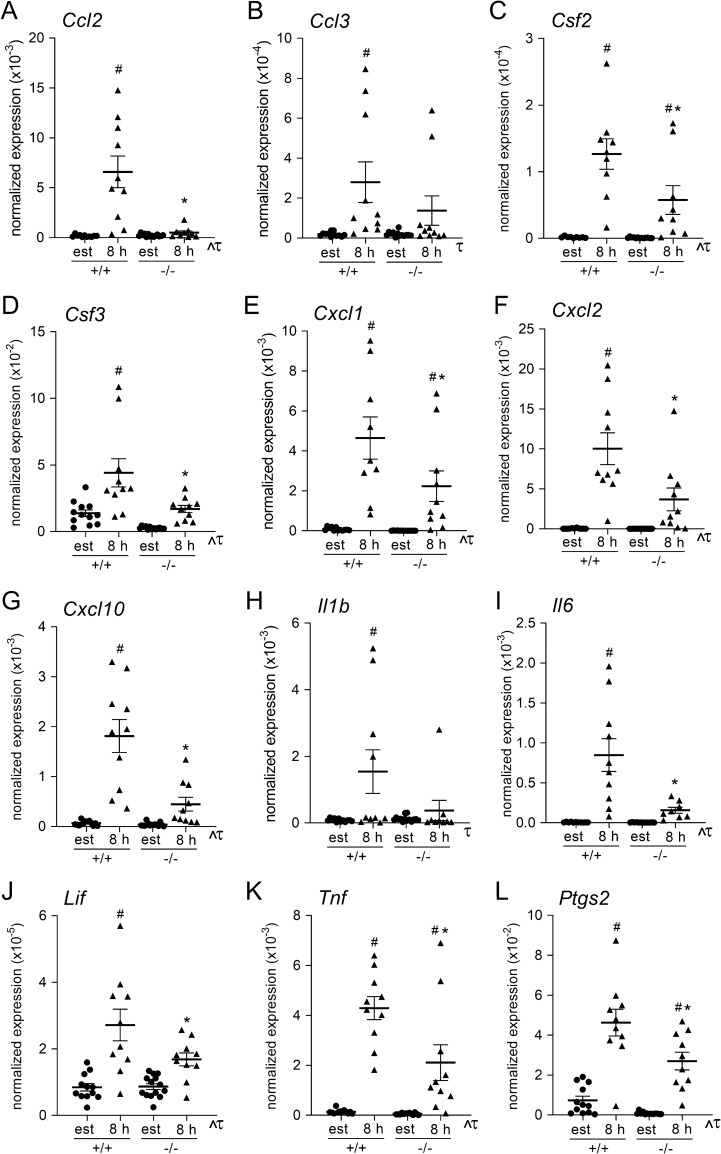
Effect of maternal TLR4 deficiency on mating-induced expression of pro-inflammatory cytokine and immune regulatory genes in the uterine endometrium. mRNA recovered from endometrial tissue of *Tlr4*^+*/*+^ (+/+) and *Tlr4*^*−/−*^ (−/−) mice at estrus, or at 8 h after mating with B6 males, was reverse transcribed into cDNA and analyzed by qPCR to quantify cytokine gene expression. Expression was normalized to *Actb* using the delta C(t) method. (**A**) *Ccl2*, (**B**) *Ccl3*, (**C**) *Csf2*, (**D**) *Csf3*, (**E**) *Cxcl1*, (**F**) *Cxcl2*, (**G**) *Cxcl10*, (**H**) *Il1b*, (**I**) *Il6*, (**J**) *Lif,* (**K**) *Tnf,* and (***L***) *Ptgs2*. Symbols depict individual mice (n = 10–14/group) and data are shown as mean ± SEM. Effects of mating and genotype were assessed by one-way ANOVA with post-hoc Sidak’s multiple comparison test (*p < 0.05 versus same time point in *Tlr4*^+/+^ females, ^#^*p* < 0.05 versus estrous control within genotype). The overall effect of genotype and mating was assessed by two-way ANOVA (τ*p* < 0.05 difference attributable to mating; ^^^*p* < 0.05 difference attributable to genotype). Data for additional cytokines *Ccl19, Ccl21*, *Il1a*, *Il10*, and *Trail,* and tSNE analysis of qPCR data is shown in Supplemental Fig. S1.

The majority of cytokines assessed showed clear evidence of induction in *Tlr4*^+/+^ females after mating. Induced cytokine genes included *Ccl2*, *Ccl3*, *Ccl19*, *Csf2*, *Csf3, Cxcl1*, *Cxcl2*, *Cxcl10*, *Il1b*, *Il6*, *Lif,* and *Tnf* (all *p* < 0.05, Fig. 2A–J,L, Supplemental Fig. S1A). In contrast, many genes were not induced after mating in *Tlr4*^*−*/*−*^ mice, including *Ccl2* (Fig. 2A), *Ccl3* (Fig. 2B), *Csf3* (Fig. 2D), *Cxcl2* (Fig. 2F), *Cxcl10* (Fig. 2G), *Il1b* (Fig. 2H), *Il6* (Fig. 2I), and *Lif* (Fig. 2J) (all *p* < 0.05). Others were induced, but to a lesser degree than in *Tlr4*^+/+^ mice, including *Csf2* (Fig. 2C), *Cxcl1* (Fig. 2E), and *Tnf* (Fig. 2K) (all *p* < 0.05). *Ptgs2*, which encodes prostaglandin-endoperoxide synthase 2 (COX2), a key regulator of prostaglandin E2 synthesis required for uterine receptivity^29^, was induced by mating but to a lesser degree in *Tlr4*^*−/−*^ mice than in *Tlr4*^+*/*+^ mice (Fig. 2L, p < 0.05). Although *Il10* was not induced after mating, it was reduced in TLR4 deficient mice (Supplemental Fig. S1D). *Il1a* and *Ccl21a* were also not induced after mating, and were not different in *Tlr4*^+/+^ versus *Tlr4*^*−*/*−*^ mice, while *Trail* was induced in *Tlr4*^*−*/*−*^ but not *Tlr4*^+/+^ mice (Supplemental Fig. S1B,C,E).

Dimensionality reduction tSNE analysis was utilized as another approach to evaluate whether TLR4 deficiency affected the pattern of cytokine response. Values from mated *Tlr4*^*−*/*−*^ and *Tlr4*^+/+^ mice for *Ccl2*, *Ccl3*, *Ccl19*, *Csf2*, *Csf3, Cxcl1*, *Cxcl2*, *Cxcl10*, *Il1b*, *Il6*, *Lif, Tnf,* and *Ptgs2* expression were concatenated and transformed into two-dimensional projections using the tSNE algorithm. Data was seen to cluster according to genotype (Supplemental Fig. S1F), supporting the interpretation that TLR4 is a determinant of uterine cytokine expression pattern in early pregnancy.

### Effect of maternal TLR4 deficiency on immune regulatory miRNAs in the uterus

**Figure 3:**
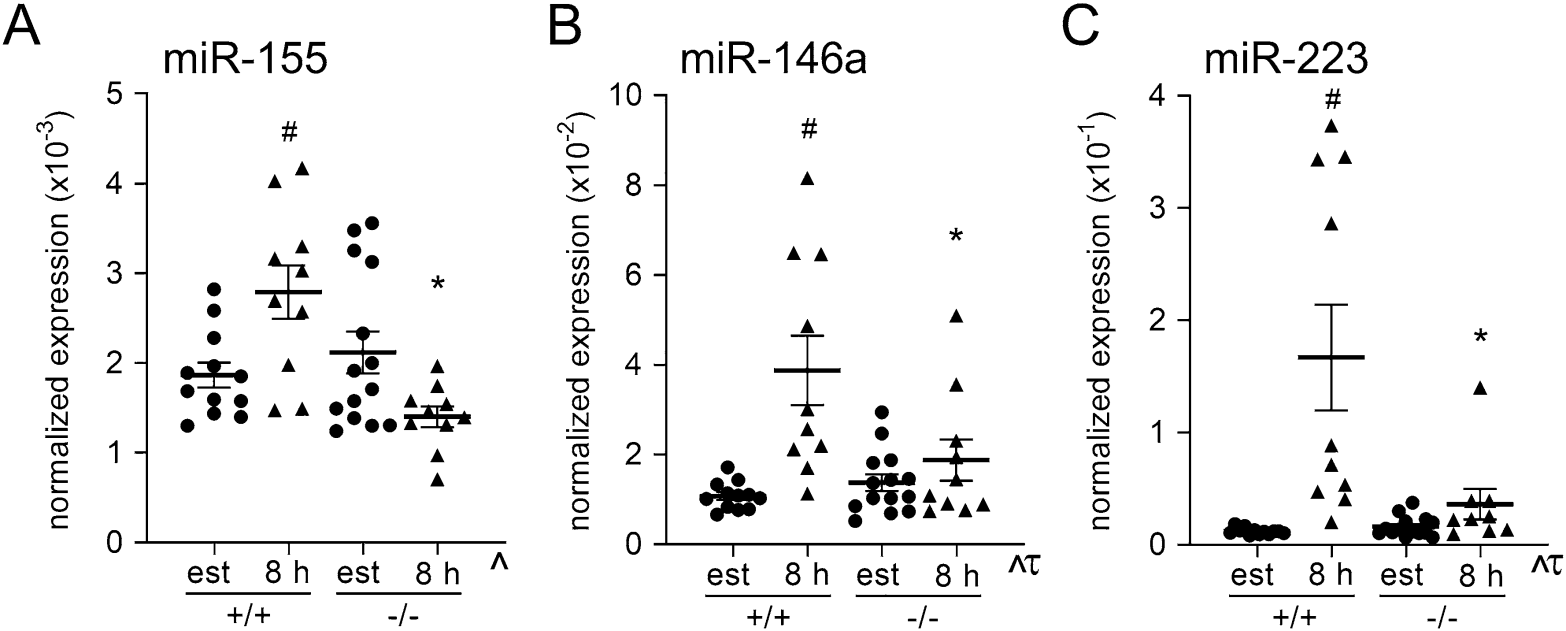
Effect of maternal TLR4 deficiency on mating-induced expression of immune-regulatory miRNAs in the uterine endometrium. miRNA recovered from endometrial tissue of *Tlr4*^+*/*+^ (+/+) and *Tlr4*^*−/−*^ (−/−) mice at estrus, or at 8 h after mating with B6 males, was analyzed by Taqman assays to quantify microRNA expression. miRNA expression was normalized to U6snRNA using the delta C(t) method. (**A**) miR-146a, (**B**) miR-155, and (**C**) miR-223. Symbols depict individual mice (n = 10–14/group) and data are shown as mean ± SEM. Effects of mating and genotype were assessed by one-way ANOVA with post-hoc Sidak’s multiple comparison test (*p < 0.05 versus same time point in *Tlr4*^+/+^ females, ^#^*p* < 0.05 versus estrous control within genotype). The overall effect of genotype and mating was assessed by two-way ANOVA (τ*p* < 0.05 difference attributable to mating; ^^^*p* < 0.05 difference attributable to genotype).

MicroRNAs miR-146a-5p (*miR-146a*), miR-155-5p (*miR-155*), and miR-223-3p (*miR-223*) are three immune-regulatory miRNAs known to be induced after TLR4 activation in other tissue settings^30^ that are potentially involved in maternal immune adaptation to pregnancy^7^. We previously showed that miR-155 is elevated in early pregnancy and is required for optimal Treg cell generation in the uterus^6^. Taqman assays were used to investigate whether maternal TLR4 alters regulation of these miRNAs during the pro-inflammatory response induced by seminal fluid. Endometrial *miR-155* expression was induced by 1.5-fold in *Tlr4*^+*/*+^ females after mating compared to estrous females (*p* = 0.023) (Fig. 3A), while *Tlr4*^*−/−*^ females did not upregulate miR-155 after mating, with 50% lower miR-155 expression compared with mated wild-type females (*p* < 0.001, Fig. 3A). Endometrial miR-146a expression was induced after mating in *Tlr4*^+*/*+^ females by 3.6-fold (*p* < 0.0001) (Fig. 3B), but not in *Tlr4*^*−/−*^ females, where expression was 52% lower than in mated *Tlr4*^+*/*+^ mice (*p* = 0.014, Fig. 3B). miR-223 showed a similar pattern with a 13.8-fold induction after mating in *Tlr4*^+*/*+^ females (*p* < 0.0001) (Fig. 3C), but no change after mating in *Tlr4*^*−/−*^ females, where expression was 78% lower than in mated *Tlr4*^+*/*+^ mice (*p* = 0.0002) (Fig. 3C).

### Effect of maternal TLR4 deficiency on Treg cell generation in early pregnancy

**Figure 4:**
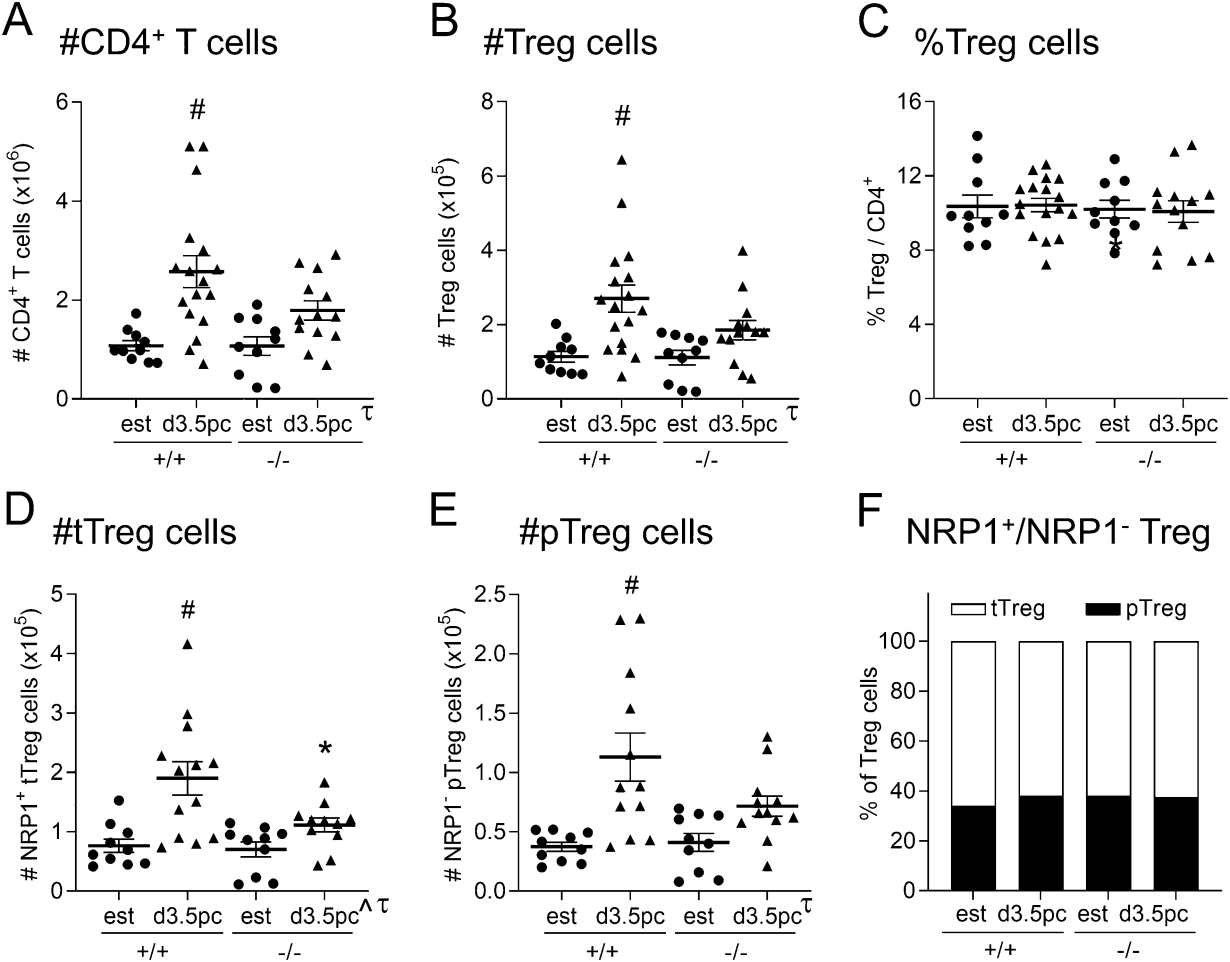
Effect of maternal TLR4 deficiency on CD4^+^, Treg, tTreg, and pTreg cells in the uterine dLN after mating. Cells harvested from the dLN of *Tlr4*^+*/*+^ (+/+) and *Tlr4*^*−/−*^ (−/−) mice at estrus, or on d 3.5 pc after mating with B6 males, were analyzed by flow cytometry to assess phenotype and numbers of CD4^+^ T cells, Treg cells (defined as CD4^+^CD25^+^FOXP3^+^), tTreg (NRP1^+^ Treg), and pTreg (NRP1^*−*^ Treg) cells. The total number of CD4^+^ T cells (**A**) and Treg cells (**B**) and the percentage of Treg cells within the CD4^+^ population were assessed (**C**). The absolute number of NRP1^+^ tTreg (**D**) and NRP1^*−*^ pTreg (**E**) cells was measured, and the proportion of NRP1^+^ tTreg (white) and NRP1^*−*^ pTreg (black) cells within the Treg cell population was calculated (**F**). Symbols depict individual mice (n = 10–17/group) and data are shown as mean ± SEM. Differences between groups were assessed by one-way ANOVA with Sidak *t* test (*p < 0.05 between genotype of the same mating status; ^#^*p* < 0.05 between estrus and mating within a genotype). The overall effect of genotype and mating was assessed by two-way ANOVA (^τ^*p* < 0.05 difference attributable to mating; ^^^*p* < 0.05 difference attributable to genotype). Gating strategy is shown in Supplemental Fig. S2, and FOXP3 mean fluorescence intensity (MFI) is shown in Supplemental Fig. S3.

The impaired pregnancy outcomes seen in *Tlr4*^*−/−*^ mice are reminiscent of effects of Treg cell deficiency^14–16^. The major site of Treg cell activation and expansion during early pregnancy is the para-aortic lymph nodes draining the uterus (dLN)^3^. The significance of TLR4 for Treg cell generation, and CD4^+^ T cell populations more broadly, was assessed by flow cytometry in the dLN of *Tlr4*^*−/−*^ and *Tlr4*^+*/*+^ control mice, on d 3.5 pc after mating (see Supplemental Fig. S2 for gating strategy). Consistent with previous studies^3,6^, mating resulted in a 2.6-fold increase in the total number of dLN CD4^+^ T cells following mating in *Tlr4*^+*/*+^ mice (*p* < 0.001) (Fig. 4A), with a 2.4-fold increase in FOXP3^+^CD25^+^ Treg cells (*p* < 0.002) (Fig. 4B). In contrast, no increase in total CD4^+^ T cells, or the FOXP3^+^CD25^+^ Treg cell subset, occurred in *Tlr4*^*−/−*^ mice after mating (Fig. 4A,B). The relative proportion of Treg cells amongst the CD4^+^ T cell population after mating was not altered by genotype (Fig. 4C).

NRP1 is a surface marker regularly used in mice to discriminate Treg cells originating in the thymus (tTreg) from peripheral Treg cells (pTreg)^31,32^. Consistent with previous studies showing NRP1^+^ Treg cells are prevalent amongst the expanding Treg cell pool in early pregnancy^12^, NRP1^+^ tTreg cells comprised ~ 60–65% of the total Treg cell population, while NRP1^*−*^ peripherally-derived pTreg constituted the remaining ~ 35–40% (Fig. 4D,E). The relative proportion of pTreg and tTreg was not impacted by genotype or mating (Fig. 4F). The total number of NRP1^+^ tTreg cells was expanded 2.4-fold in *Tlr4*^+*/*+^ mice compared to estrous controls (*p* < 0.001) (Fig. 4D), but there was no comparable expansion in *Tlr4*^*−/−*^ mice (Fig. 4D), so that tTreg cells were ~ 40% fewer in mated *Tlr4*^*−/−*^ mice compared to mated *Tlr4*^+*/*+^ mice (*p* = 0.014). A similar response was evident in dLN NRP1^*−*^ pTreg cells, where mating boosted pTreg cell numbers by 3.0-fold in *Tlr4*^+*/*+^ mice (Fig. 4E, p < 0.001), but did not elicit an increase in *Tlr4*^*−/−*^ mice. TLR4 deficiency did not impact the amount of FOXP3 expression in total Treg, tTreg, or pTreg cells after mating, as assessed by FOXP3 MFI (Supplemental Fig. S3).

**Figure 5:**
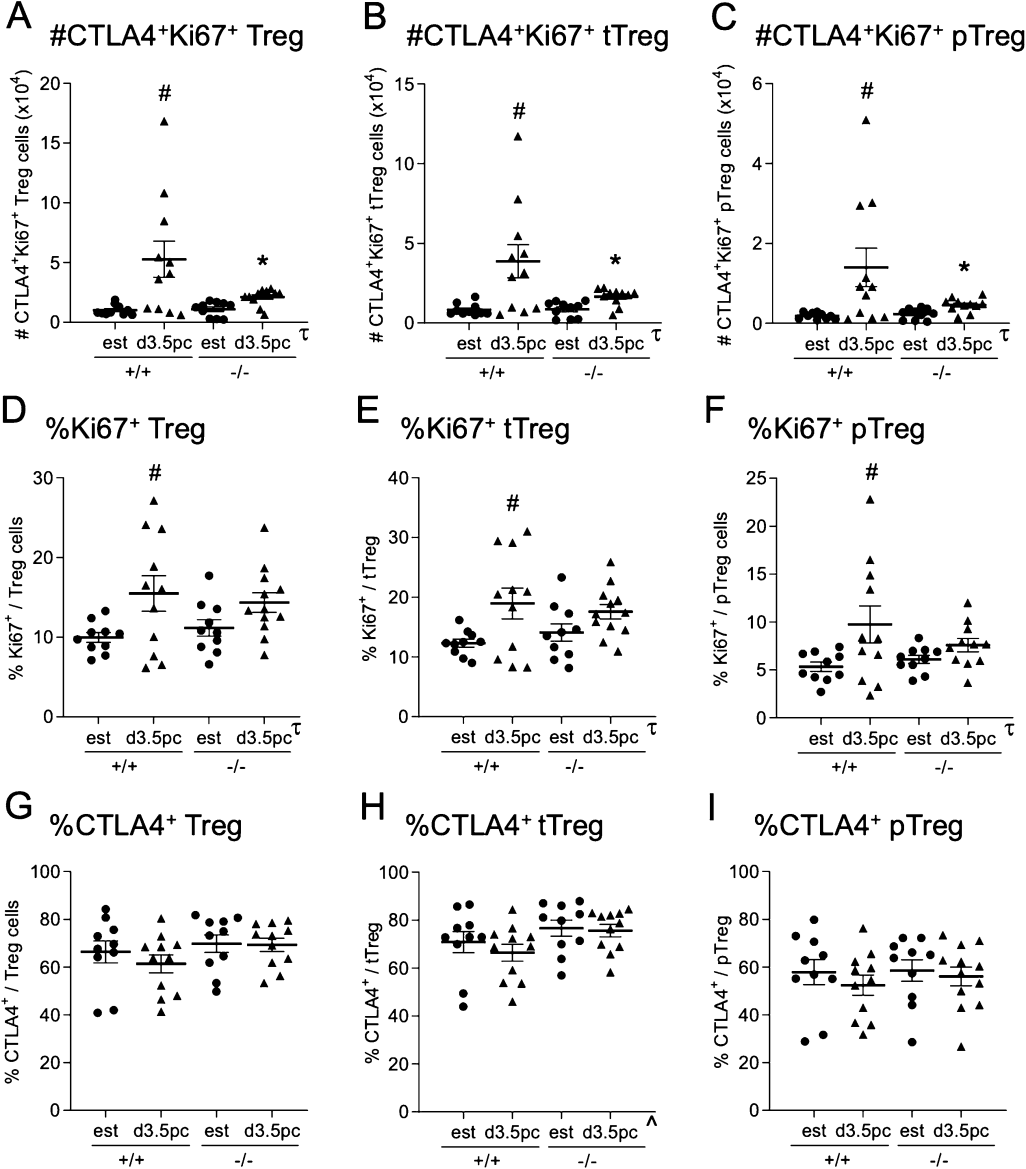
Effect of maternal TLR4 deficiency on expression of CTLA and Ki67 by Treg cells in the uterine dLN after mating. Cells harvested from the dLN of *Tlr4*^+*/*+^ (+/+) and *Tlr4*^*−/−*^ (−/−) mice at estrus, or on d 3.5 pc after mating with B6 males, were analyzed by flow cytometry to assess expression of the suppressive marker CTLA4 and proliferation marker Ki67 in Treg cells (defined as CD4^+^CD25^+^FOXP3^+^) and tTreg (NRP1^+^ Treg) and pTreg (NRP1^*−*^ Treg) cells. The total number of double positive CTLA4^+^Ki67^+^ Treg (**A**), NRP1^+^ tTreg (**B**) and NRP1^*−*^ tTreg (**C**) cells was quantified. The proportion of Ki67^+^ cells within the total Treg (**D**), NRP1^+^ pTreg (**E**) and NRP1^*−*^ tTreg (**F**) cell populations, and the proportion of CTLA4^+^ cells within the total Treg (**G**), NRP1^+^ pTreg (**H**) and NRP1^*−*^ tTreg (**I**) cell populations, was determined. Symbols depict individual mice (n = 10–17/group) and data are shown as mean ± SEM. Differences between groups were assessed by one-way ANOVA with Sidak *t* test (*p < 0.05 between genotype of the same mating status, ^#^*p* < 0.05 between estrus and mating within a genotype). The overall effect of genotype and mating was assessed by two-way ANOVA (τ*p* < 0.05 difference attributable to mating; ^^^*p* < 0.05 difference attributable to genotype).

To assess whether TLR4 deficiency impacted Treg cell phenotype in the peri-implantation phase, the suppressive marker CTLA4^33^ and the marker of proliferation Ki67 were analyzed in dLN Treg, NRP1^+^ tTreg and NRP1^*−*^ pTreg populations. Compared to estrous mice, mating induced a 5.1-fold increase in the absolute number of Treg cells expressing both CTLA4 and Ki67 (*p* < 0.003) (Fig. 5A) in *Tlr4*^+*/*+^ mice, a subset we showed previously to be selectively increased after mating^6,12^. No increase was seen in response to mating in *Tlr4*^*−/−*^ mice, where CTLA4^+^Ki67^+^ Treg cells were 63% fewer than in mated *Tlr4*^+*/*+^ mice (*p* < 0.030) (Fig. 5A). A similar pattern was seen in both the NRP1^+^ tTreg (Fig. 5B) and NRP1^*−*^ pTreg (Fig. 5C) subpopulations. Elevated expression of Ki67 was most notable, such that in *Tlr4*^+*/*+^ mice, mating induced a 1.6-fold increase in the proportion of proliferating Treg cells positive for Ki67 (*p* = 0.044) (Fig. 5D), evident in both the NRP1^+^ tTreg (Fig. 5E) and NRP1^*−*^ pTreg (Fig. 5F) cells. In contrast, *Tlr4*^*−/−*^ mice did not exhibit any change in Ki67 in total Treg cells (Fig. 5D), NRP1^+^ tTreg cells (Fig. 5E), or NRP1^*−*^ pTreg cells (Fig. 5F) after mating. The proportion of total Treg (Fig. 5G), NRP1^+^ tTreg (Fig. 5H), and NRP1^*−*^ pTreg (Fig. 5I) expressing CTLA4 was not changed after mating and not impacted by TLR4 deficiency.

**Figure 6:**
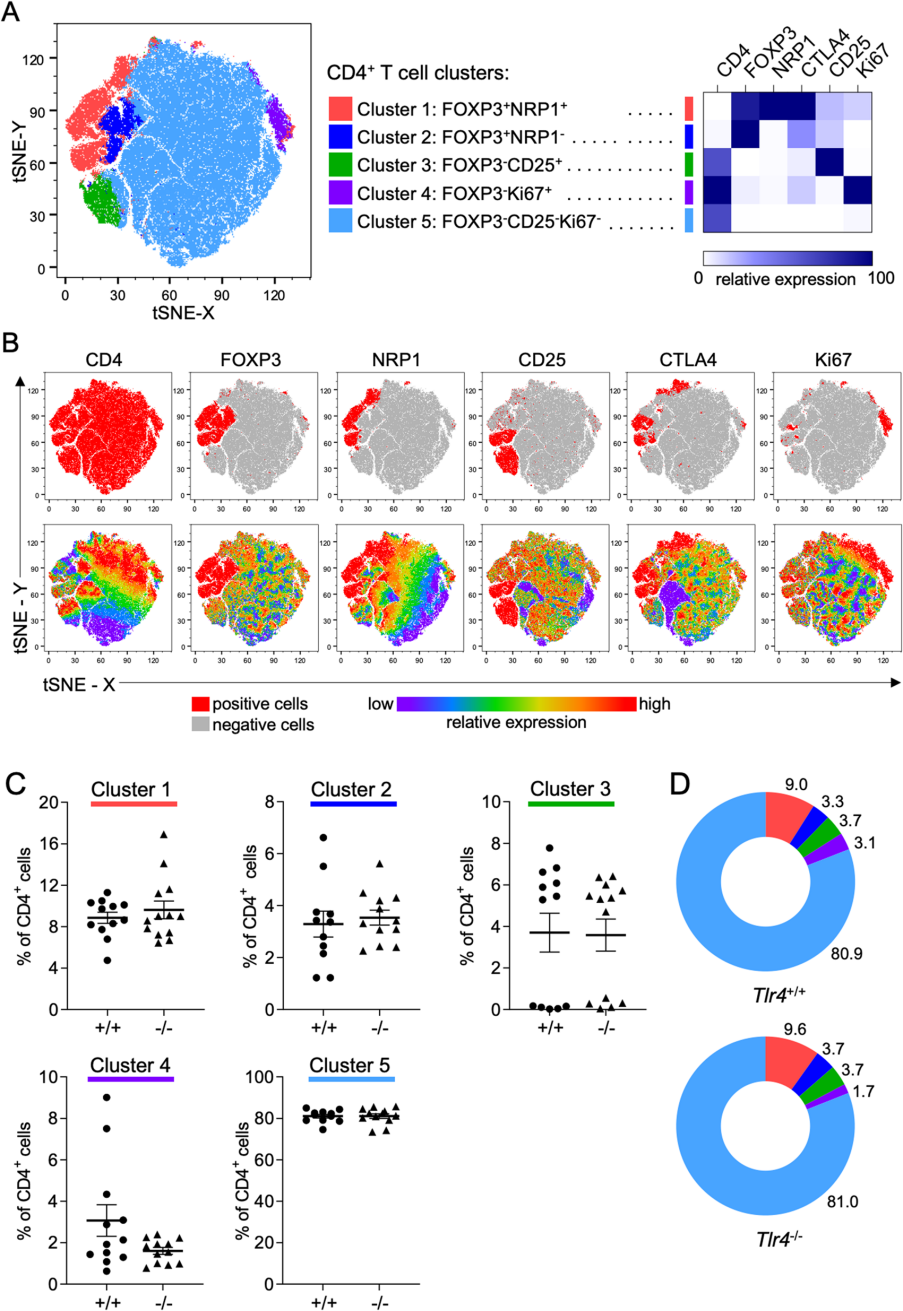
Assessment of the CD4^+^ T cell population by dimensionality-reducing tSNE algorithm. CD4^+^ T cells from the uterine dLN of *Tlr4*^+/+^ (+/+) or *Tlr4*^*−*/*−*^ (−/−) mice on d 3.5 pc after mating with B6 males were analyzed by flow cytometry. The viable CD4^+^ T cells from all samples were concatenated into a single file and transformed by the tSNE algorithm. X-shift identified 5 unique cell clusters (**A**), and the relative expression intensity of each of CD4, FOXP3, NRP1, CD25, CTLA4, and Ki67 within each cluster was calculated and shown by a heat map (**A**). Cells positive for each marker are shown in red on the tSNE (**B**—top row), and the relative expression of each marker is also shown (**B**—bottom row). The percentage of cells comprised by each of the 6 clusters for the d 3.5 pc groups in *Tlr4*^+/+^ and *Tlr4*^*−*/*−*^ mice was calculated. Symbols depict individual mice (n = 8–13/group) and data are also shown as mean ± SEM (**C**). Effects of genotype were assessed by unpaired Sidak *t* test. No effects of genotype were found. The contribution of each cluster to the tSNE in *Tlr4*^+/+^ and *Tlr4*^*−*/*−*^ mice is shown as pie charts (**D**).

tSNE analysis was utilized to gain insight on whether TLR4 deficiency differentially impacted Treg cell generation in the dLN after mating. Flow cytometry data on dLN CD4^+^ T cells from mated *Tlr4*^*−*/*−*^ and *Tlr4*^+/+^ mice stained for CD4, FOXP3, CD25, NRP1, CTLA4, and Ki67 were concatenated and transformed into two-dimensional projections using the tSNE algorithm. Inspection of density and contour plots revealed 5 distinct clusters, amongst which subpopulations of FOXP3^+^CD4^+^ T cells with the characteristics of tTreg and pTreg cells could be located (Fig. 6A,B). There was no difference in the relative proportions of different FOXP3^+^ or FOXP3^*−*^ clusters attributable to TLR4 deficiency (Fig. 6C,D). This confirms there was no substantial differential impact of TLR4 deficiency within the Treg cell compartment, and that fewer Treg cells after mating in *Tlr4*^*−*/*−*^ mice reflects an impaired response in the whole CD4^+^ T cell population.

### Effect of maternal TLR4 deficiency on innate immune cells in early pregnancy

The reduced T cell response seen in *Tlr4*^*−/−*^ mice could reasonably be the consequence of reduced recruitment of innate immune cells into the uterus, since these cells are required for antigen presentation and influence the phenotype of T cells generated in the adaptive immune response. Macrophages, dendritic cells, and neutrophils are abundant in the uterus and each participate in the immune response to seminal fluid^22,34^. Previously, we showed that the number of neutrophils recruited into the endometrium after mating is substantially less in *Tlr4*^*−*/*−*^ mice than in *Tlr4*^+/+^ mice^22^. To determine whether TLR4 is also required for macrophage and dendritic cell recruitment into the uterine endometrium, uteri were recovered from *Tlr4*^*−*/*−*^ and *Tlr4*^+/+^ females mated with intact wild-type males, then macrophages (F4/80^+^ cells) and dendritic cells (MHC class II^+^ cells) were detected by immunohistology. Macrophages were recruited into the uterine endometrium and myometrium at 8 h after mating in both *Tlr4*^*−*/*−*^ and *Tlr4*^+/+^ females (Supplemental Fig. S4A), and the relative abundance was not dependent on genotype in either tissue compartment (Supplemental Fig. S4B,C). Similarly, an influx of dendritic cells was evident in both the endometrium and myometrium after mating in both *Tlr4*^*−*/*−*^ and *Tlr4*^+/+^ females (Supplemental Fig. S5A), to a degree that was not affected by genotype (Supplemental Fig. S5B,C). This indicates that unlike neutrophils, TLR4 is not essential for seminal fluid-mediated attraction of macrophages or dendritic cells into the endometrium tissue, and suggests that of the three innate immune cell lineages, altered neutrophils might contribute to the reduced T cell response in the absence of TLR4.

## Discussion

Events in the pre-and peri-implantation phase are critical for generating maternal immune tolerance to allow successful embryo implantation and progression of healthy pregnancy^2,35,36^. Fetal loss, fetal growth restriction, and preeclampsia and related hypertensive pregnancy disorders, are all thought to originate around the time of embryo implantation, due to insufficient placental invasion and impaired access to the maternal blood supply^37^. In the mouse, immune adaptation to accommodate invading cells of the semi-allogeneic conceptus commences with a controlled inflammatory response provoked by seminal fluid in the pre-implantation phase. Recently, we employed microarray and reverse bioinformatics to identify pathways that are activated in the uterus by seminal fluid contact^21^. TLR4 expressed by uterine epithelial cells was implicated as a key signaling mediator that ligates moieties carried by sperm, as well as soluble factors in seminal plasma, to induce cytokine and chemokine expression^22^. The current study provides compelling evidence that TLR4 has an essential role in maternal immune adaptation in early pregnancy. We demonstrate that an impaired peri-conception inflammatory response due to absence of TLR4 signaling acts to constrain the generation of adequate Treg cells, and profoundly disrupt the normal trajectory of fetal growth and development.

The fetal demise observed in the absence of TLR4 mostly occurred in mid-gestation, with fetal resorption rate increased around fivefold in *Tlr4*^*−/−*^ compared to wild-type females. The size of the residual hematogenous mass evident at autopsy in late gestation indicates loss around d 10–12 pc, a critical point in mouse fetal development just after transition from yolk sac nutrition to dependence on a functional placenta occurs, when deficiency in placental development is often revealed^38^. Typically, this is a vulnerable time when embryos can easily succumb to oxidative stress secondary to poor decidual vascular remodelling^39^, which can be exacerbated by insufficient Treg cells^13^ or imbalance in the T cell response leading to excess T effector cells^40,41^. Fetal growth restriction typically also originates before mid-gestation due to suboptimal placental development^38^. This points to an impact of TLR4 deficiency in the first half of gestation, and most likely during implantation and early placental morphogenesis.

A causal relationship between the perturbed immune environment during the peri-conception period of pregnancy and later fetal loss and fetal growth restriction in TLR4 deficient mice is biologically rational and supported by several previous studies that show the critical importance of immune cells for placental morphogenesis. Most importantly, Treg cells are required for the increased uterine vascular dilatation that accompanies implantation, without which mid-gestation embryo resorption commonly occurs^13^. Given the importance of Treg cells in uterine artery remodelling, placental morphogenesis, and fetal growth, it seems probable that dysregulated Treg cell function in *Tlr4*^*−/−*^ females contribute to the fetal phenotype observed in this study. However, whether absence of Treg cells is the only or most critical causal factor remains to be determined.

This study confirms and expands on our earlier report that TLR4 is essential for the full induction of key immune regulatory genes in the endometrium responding to seminal fluid-mediated stimulation during the peri-conception phase. We previously showed that *Csf3*, *Cxcl2*, *Cxcl10*, *Il6*, *Lif*, and *Tnf* are differentially regulated in the uterus of *Tlr4*^*−*/*−*^ mice^21^, and each of these were confirmed in the current study. We now demonstrate that *Ccl2, Ccl3, Csf2, Cxcl1, Il1b*, and *Ptgs2* are also either not induced, or induced to a lesser degree, when TLR4 is absent. These data are consistent with TLR4 ligands present in semen being important upstream drivers of TLR4 activation in endometrial cells. Our recent microarray analysis showed that sperm, as well as seminal plasma, contribute to induction of TLR4-regulated cytokines in the endometrium^22^. Using in vitro cultures of uterine epithelial cells recovered from *Tlr4*^*−*/*−*^ and *Tlr4*^+/+^ mice, we showed that epithelial cell TLR4 is required to elicit sperm-mediated induction of IL6, CSF3, and CXCL2, and that several other cytokines released from epithelial cells were dysregulated in the absence of TLR4^22^. Immune cells residing in the female reproductive tract also express TLR4 and may respond directly to TLR4-mediated stimulation to contribute to the observed differential cytokine expression. Several TLR4 ligands exist in seminal fluid including pathogen-associated molecular patterns (PAMPs) such as bacterial lipopolysaccharide (LPS), and endogenous damage-associated molecular patterns (DAMPs), including β-defensins and heat shock proteins^4^. While bacterial LPS is detectable in seminal fluid, the amount detected is insufficient to explain endometrial cytokine induction after mating^21^, suggesting endogenous DAMPs in seminal fluid are responsible. Sperm carry several DAMPs on the surface including β-defensins^42^ and hyaluronan fragments^43^ with potential to bind TLR4 in the female reproductive tract.

Many of the cytokines and chemokines induced by TLR4 signaling act to regulate influx of innate immune cell populations into the endometrium in the inflammation-like response to mating^9,10,36^. If these innate immune cells were dysregulated in TLR4-deficient mice, this would provide a mechanism linking TLR4 signaling with impaired Treg cell generation. In particular, dendritic cells are critical determinants of the quality of a T cell response^23,24^ and act by presenting seminal fluid antigens to stimulate Treg cells activation and proliferation^2,44^. Others have demonstrated that TLR4 signaling is required for Treg cell proliferation, through mechanisms involving effects on innate immune cells. For example, *Tlr4*^*−*/*−*^ mice infected with *Fusobacterium nucleatum* exhibit a reduced T cell response to bacterial antigens and Treg cells are differentially impacted^45^. It has been shown that low dose LPS induces tolerogenic Treg cell skewing through a TLR4-dependent mechanism, associated with tolerogenic DC induction^46^, while TLR4 is required for the post-traumatic induction of Treg cells in a murine burn injury model^47^.

The uterine genes dysregulated by TLR4 deficiency include *Ccl2*, *Csf2*, and *Tnf*, the cytokine products of which regulate the dynamics and phenotype of dendritic cells^48,49^. Prostaglandin E_2_ (PGE_2_) synthesis regulated by *Ptgs2* expression enhances the maturation, migration and antigen-presenting capacity of DCs, and is a major driver of pro-tolerogenic phenotype through induction of the tryptophan catabolizing enzyme indoleamine 2,3-dioxygenase (IDO)^50^. PGE_2_ is a critical microenvironmental factor linked with Treg cell generation^18^ and especially tTreg functional competence^51^, and is essential for acquisition of endometrial receptivity to embryo implantation^29^. Thus, we reasoned that insufficient DCs might contribute to the reduced CD4^+^ T cell and Treg cell response seen in the absence of TLR4^23,24^. Surprisingly, we observed that the number of MHC class II^+^ DCs were not changed in *Tlr4*^*−*/*−*^ dams after mating. However, given the known responsiveness of DCs to TLR4-regulated cytokines^48,49^, it seems likely that uterine DCs in TLR4-deficient mice could have impaired antigen presenting function, but further studies are required to confirm this.

Monocyte-derived macrophages are another prominent leukocyte population recruited into the uterus before implantation^10^ that potentially contribute to priming T cells in early pregnancy^34^. Reasonably, macrophage recruitment was also expected to be impacted by TLR4 ablation^26^, since TLR4-regulated chemokines CCL2 and CCL3, cytokines CSF2, IL6, and LIF, and PGE_2_ regulated by *Ptgs2* expression, are all implicated in regulating uterine macrophages in early pregnancy^9,10^. Nevertheless we did not observe any change in the relative abundance of F4/80^+^ cells recruited after mating, although F4/80^+^ cells may have an altered phenotype due to the absence of TLR4.

Neutrophil recruitment and activation is regulated by several cytokines and chemokines induced by TLR4 signaling^26,52^. We recently reported that neutrophil recruitment into the uterine mucosa and lumen after mating depends on sperm-associated signals that interact with the uterus through ligating TLR4 to induce *Cxcl2, Il6,* and *Csf3*^21,22^. In the hours after mating neutrophils are recruited into the endometrial tissue immediately underlying the luminal epithelium, and many enter the uterine lumen where they remove superfluous sperm, microbes and debris after mating^8^. A lack of endometrial neutrophils in *Tlr4*^*−/−*^ females is therefore a strong candidate mechanism for contributing to the ensuing Treg cell deficiency, given that Treg cells with an angiogenic phenotype have been shown to be neutrophil-dependent and required for normal placental development^28^. Another TLR4-induced cytokine gene *Cxcl10* may be involved in recruitment and positioning of Treg cells in the uterus^53^, although *Ccl19*, which we showed previously to be regulated by seminal fluid and to influence Treg cell recruitment^54^, was not impacted by TLR4 deficiency.

This study also provides evidence that maternal TLR4 contributes to the regulation of the female post-mating immune response by post-transcriptional mechanisms involving miRNA regulation. Previous reports have shown that TLR4 is involved in the upregulation of *miR-146a*, *miR-155* and *miR-233* in various tissues^30^. Notably we have recently reported that miR-155 is essential for induction of the T cell response to pregnancy, and its deficiency differentially impacts generation of uterine Treg cells of both the tTreg and pTreg compartments^6^. Here, we showed that maternal TLR4 is required to induce *miR-155* as well as *miR-146a* and *miR-233* miRNAs in the endometrium after mating. These three miRNAs are highly expressed and exert a range of effects in the immune system, particularly in Treg cells, DCs and macrophages, all of which are contribute to establishing pregnancy tolerance^55^. While other TLRs, including TLR2 and TLR3 have also been linked with induction of these miRNAs in other tissue settings^30^, our data shows that in the uterine context, TLR4 plays an indispensable role in their induction. These three miRNAs are all involved in regulation of the NF-κB inflammatory pathway that is activated by TLR4 ligation^56^. Given that *Cxcl2*, *Cxcl10*, *Il6,* and *Tnf* are also regulated by NF-κB and are dysregulated in the uterus in the absence of maternal TLR4, these findings imply that a key function of TLR4 is to induce miRNAs that regulate cytokine gene expression via activation of NF-κB.

Cytokines induced by TLR4 also have potential to support or suppress fetal and placental development through programming effects on the pre-implantation embryo. CSF2, CSF3, LIF, and IL6 have each been implicated as embryotrophic growth factors that boost implantation competence and program healthy fetal and placental development^57–59^. Absence of embryo contact with CSF2 before implantation alters placental development, elevating rates of fetal growth restriction and fetal death^60^. Thus, failure of induction of embryotrophic factors may also contribute to poor outcomes in later gestation in TLR4 deficient females. Elevated *Trail* expression in the absence of maternal TLR4 would be predicted to decrease embryo quality, and adversely impact fetal development in utero^58,61^. Failure of mated *Tlr4*^*−/−*^ females to attenuate *Trail* induction may impact blastocysts at implantation and contribute to setting a course of altered fetal and placental development that leads to later fetal loss.

As well as uterine epithelial cells, TLR4 is expressed by many of the uterine leukocyte lineages involved in implantation. Macrophages are activated in response to endogenous and microbial TLR4 ligands to release pro-inflammatory cytokines and acquire M1 polarisation through NF-κB activation^25^, and TLR4 null mutation disposes macrophages towards an M2 phenotype^62^. Macrophages have been implicated in uterine receptivity through regulating glycosylated structures that regulate embryo attachment to uterine epithelial cells^63^, and by modulating angiogenesis and decidual vascular adaptation^64^. It seems unlikely that reduced M1 macrophage activation directly accounts for the fetal phenotype in TLR4 deficient mice, as generally their pro-implantation effects are associated with an M2 phenotype, and M1 macrophages are viewed as detrimental to immune tolerance and implantation success^65^. Nevertheless, some functions of M2 macrophages may depend on macrophage sensing via TLR4, as demonstrated for release of TGFB to support generation of Treg cells in schistosome-infected mice^66^, On the other hand, dendritic cells acquire maturation markers in direct response to TLR4 ligands and can attain either a pro-tolerogenic or immunogenic phenotype^67^, depending on the cytokine microenvironment including CSF2, CSF3, TNF and IL10^68,69^. TLR4 is also abundantly expressed by the fetal trophoblast and other placental lineages^70^, but in the current mating combination the fetus and placenta are heterozygous for *Tlr4* null mutation and would express TLR4.

TLR4 signaling in mid- to late gestation appears unnecessary for normal fetal growth, since administration of TLR4 antagonist (+)-naloxone does not impair healthy neonatal and adult outcomes^71^. The major role of TLR4 in late gestation is to sense maternal and fetal triggers that initiate parturition and affect the timing of birth^72^. A detrimental impact of genetic TLR4 deficiency in later gestation would not explain the fetal phenotype seen herein, given that fetal loss clearly occurred early, and several other studies show TLR4 activation by microbial or endogenous factors after mid-gestation is generally linked with adverse outcomes. As early as day 10 of gestation, administration of low dose LPS causes fetal growth restriction and fetal loss^73,74^, and after day 15, LPS or endogenous TLR4 ligands such as platelet activating factor or HMGB1, cause fetal loss and preterm birth^75–77^. Furthermore TLR4 signaling exacerbates placental inflammatory pathology in the event of local infection^78^.

Fetal growth restriction in TLR4 deficient mothers was accompanied by an increase in placental size, and a decrease in fetal to placental weight ratio. Fetal to placental weight ratio is commonly used as an indicator of placental transport efficiency and elevation in this parameter is a common response reflecting adaptive compensation for insufficient maternal vascular supply^79^. A previous study utilising C3H/He mice, which have a natural mutation in *Tlr4*, also reported fetal growth restriction and structural changes to the placenta, along with greater fetal loss after impaired capacity to respond to uterine ischaemia/reperfusion injury^80^. Compared with TLR4 replete C3HOuJ mice, fetal weight in C3H/He mice was reduced in late gestation to an extent similar to the current findings, while placental morphology analysis showed prominent pathophysiological changes, attributed to calcification in the placental labyrinth zone^80^. We also found changes in the placentas of *Tlr4*^*−*/*−*^ dams, with a notable increase in the relative size of the placental junctional zone accounting for the elevated placental weight—a structural anomaly that could reflect impaired placental transport function to result in FGR—but no overt changes to the placental labyrinth zone. The different impact of maternal TLR4 deficiency on placental structure between the two studies may be related to the different mating strategies employed. In the current study, the fetal placental unit was hemizygous for the *Tlr4* null mutation and MHC disparate to the mother, whereas the earlier study utilized males of the same strain to generate fetal placental units that were homozygous for the *Tlr4* null mutation but MHC identical to the dam, and so less dependent on Treg cells for maternal fetal tolerance^1,2^.

In summary, this study illustrates the importance of maternal TLR4 in initiating successful pregnancy through activation of the female immune response in the peri-conception period. Specifically, TLR4 is essential for induction of several endometrial cytokines and miRNAs after mating, which in turn drive priming of T cells and expansion of the Treg cell pool. This study expands knowledge of the role of TLR4 in pregnancy, showing that TLR4 signaling is permissive and beneficial for early pregnancy, in contrast to its adverse effects leading to fetal loss and preterm birth in later gestation^73–78^. It remains to be determined whether impaired TLR4 signaling, perhaps as a consequence of altered seminal fluid composition in men, or dysregulated TLR4 expression or *TLR4* gene polymorphisms in women, contributes to fertility or pregnancy disorders in the clinical setting.

## Materials and methods

### Mice

BALB/c females and C57Bl/6 male mice were purchased from the University of Adelaide Central Animal Facility and Animal Resources Centre (Perth, Australia) respectively. Mice with a null mutation in *Tlr4* (*Tlr4*^*−/−*^) back-crossed to BALB/c mice for more than 10 generations were sourced from Professor Shizuo Akira (University of Osaka, Osaka, Japan) and supplied by Professor Paul Foster (University of Newcastle, New South Wales, Australia). All mice used in this study were provided food and water ad libitum and housed under a 12L/12D cycle in a pathogen-free facility of Laboratory Animal Services (LAS) at the University of Adelaide, South Australia. All experiments adhered to the ARRIVE guidelines (https://arriveguidelines.org), were approved by the University of Adelaide Animal Ethics Committee (ethics approval numbers M/2014/023 and M/2017/006), and were performed in accordance with the relevant guidelines and regulations**.**

### Mating and tissue collection

Mouse estrous cycle was monitored by the examination of vaginal lavage cytology as previously described^81^. Wild-type *Tlr4*^+*/*+^ BALB/c or *Tlr4*^*−/−*^ females at 8–14 weeks of age were housed 1:1 with proven fertile C57Bl/6 stud males at 2330–0100 h on the night of proestrus detection. The time of mating was monitored by infrared video recording and confirmed by the presence of a copulatory plug the following morning (designated d 0.5 pc).

For gene expression and immunohistological analysis, females were humanely killed at 8 h pc by cervical dislocation and the uterus was excised. Unmated estrous control females were humanely killed and the uteri were excised at 2200–2300 h on the evening of proestrus detection. Fat, mesentery and blood vessels were removed under a dissection microscope (Olympus, New York, NY), then transferred in fresh PBS and slit lengthwise. To recover RNA and miRNA, endometrial tissue was scraped off using a razor blade (ProSciTech, Queensland, Australia) into 700 μl of Qiazol lysis reagent (Qiagen, Victoria, Australia), followed by homogenisation by Precellys homogenizer (Bertin, France) at a setting of one cycle at 5000 rpm for 15 s. For immunohistological assessment of immune cells, uteri were fixed in 4% paraformaldehyde (wt/vol) in PBS overnight at 4 °C, washed in PBS, and stored in 70% ethanol at 4 °C before embedding in paraffin wax.

For analysis of pregnancy outcomes, mated *Tlr4*^+*/*+^ BALB/c and *Tlr4*^*−/−*^ females were killed by cervical dislocation at 1000–1200 h on d 17.5 pc. Intact uteri were excised and the number of viable and resorbing implantation sites were counted. Each viable fetus and placenta was dissected and weighed. Two placentas were randomly chosen from each pregnant dam and fixed in 4% paraformaldehyde (wt/vol) in PBS. After overnight fixation, placentas were bisected in the midline from maternal to fetal sides to yield mid-sagittal faces and fixed for a further 3 h, then washed in PBS, and stored in 70% ethanol at 4 °C before embedding in paraffin wax.

### Isolation of RNA

Total RNA was extracted from endometrium using miRNeasy RNA extraction and purification kits (Qiagen), and treated with TURBO DNA-free kit (Life Technologies) according to the manufacturer’s instructions, then stored at − 80 °C. Successful genomic DNA removal was confirmed by quantitative-polymerase chain reaction (qPCR) using genomic-DNA specific primers (Table 1). RNA concentration was quantified using a NanoDrop Spectrophotometer ND-1000 (Thermo Fisher Scientific, Scoresby, Australia). RNA integrity was assessed by RNA Agilent Bioanalyzer (Agilent Technologies, Santa Clara, CA) to ensure all samples showed an RNA integrity number of > 7.

**Table 1:** Primers and target mRNA for qPCR analysis. 5′ represents forward primer sequence and 3′ represents reverse primer sequence. Sequences are from 5′ to 3′.

Target gene	Primer sequences	Conc (μm)	GeneBank
*Actb*	5′-TGTGATGGTGGGTATGGGTC	0.25	NM_007393
	3′-ACACGCAGCTCATTGTA		
*Ccl2*	5′-CCAGCAAGATGATCCCAATGA	0.25	NM_011333
	3′-TCTCTTGAGCTTGGTGACAAAAAC		
*Ccl3*	5′-TGCCCTTGCTGTTCTTCTCTG	0.25	NM_011337
	3′-AACGATGAATTGGCGTGGA		
*Ccl19*	5′-TGCTGGTTCTCTGGACCTTCC	0.25	NM_011888
	3′-ACGATGTTCCCAGGGATGG		
*Ccl21a*	5′-TCCAACTCACAGGCAAAGAGG	0.25	NM_011124
	3′-GCAGATGTGATGGTTGAAGCA		
*Cd14*	5′-GCTTGGCTTGTTGCTGTTGC	0.25	NM_009841
	3′-TCGGATCTGAGAAGTTGCAGG		
*Csf2*	5′ –CCTGGGCATTGTGGTCTACAG	0.05	NM_009969
	3′-GGCATGTCATCCAGGAGGTT		
*Csf3*	5′-GCAGACACAGTGCCTAAGCCA	0.25	NM_009971
	3′-CATCCAGCTGAAGCAAGTCCA		
*Cxcl1*	5′-ATTGTATGGTCAACACGCACG	0.5	NM_008176
	3′-TTTGAACGTCTCTGTCCCGAG		
*Cxcl2*	5′-GAACTGCGCTGTCAATGCCT	0.5	NM_009140.2
	3′-CCGCCCTTGAGAGTGGCTAT		
*Cxcl10*	5′-TTCATCACTCCCCTTTAGCCA	0.25	NM_021274.2
	3′-TGTCTCAGGACCATGGCTTGA		
*Il1a*	5′-CCGACCTCATTTTCTTCTGG	0.25	NM_010554.4
	3′-GTGCACCCGACTTTGTTCTT		
*Il1b*	5′-CCCAAGCAATACCCAAAGAA	0.25	NM_008361.3
	3′-GCTTGTGCTCTGCTTGTGAG		
*Il6*	5′-ACAACCACGGCCTTCCCTAC	1.0	NM_031168
	3′-TCCACGATTTCCCAGAGAACA		
*Il10*	5′-AGGCGCTGTCATCGATTTCT	0.25	NM_010548.2
	3′-TGGCCTTGTAGACACCTTGGT		
*Lif*	5′-CGCCAATGCTCTCTTCATTTC	0.5	NM_008501
	3′-TCCGATGCTCCACCAACT		
*Ptgs2 *(*Cox2*)	5′-GTTTGCATTCTTTGCCCAGC	0.25	NM_011198.3
	3′-AGTCCACTCCATGGCCCAGT		
*Tnf*	5′-GTAGCCCACGTCGTAGCAAAC	1.0	NM_013693
	3′-CTGGCACCACTAGTTGGTTGTC		
*Tnfsf10 *(*Trail*)	5′-CCAGAGATGCCGAGTACGGA	0.5	NM_009425.2
	3′-AAGGCTCCAAAGAAGCTGGCT		
*gDNA*	5′-GGCACTGACTGAGGT	0.25	N/A
	3′-GTCACAATCACAGAG		

### mRNA and miRNA expression analysis

For mRNA expression analysis, total DNase-treated RNA was reversed transcribed into cDNA from 800 ng RNA primed with 50 ng random hexamers using Superscript-IV Reverse Transcription kit (Life Technologies) according to the manufacturer’s instruction and stored at − 20 °C. Primer pairs specific for published cDNA sequences were designed using Primer Express version 2 software (Life Technologies; Table 1), optimized as previously described^5^. qPCR was performed using 20 ng cDNA, PCR primers (Table 1) and 1 × PowerUp SYBR Green PCR master mix (Thermo Fisher Scientific) employing the QuantStudio 3 Real-Time PCR System (Thermo Fisher Scientific) using the following conditions: 50 °C for 2 min, 95 °C for 2 min, and 40 cycles of 95 °C for 1 s followed by 60 °C for 30 s. The delta C(t) method was used to calculate mRNA abundance normalized to *Actb* expression^82^.

For miRNA expression analysis, hsa-miR-146a (ID 000468), mmu-miR-155 (ID 002571), hsa-miR-223 (ID 002295) and U6snRNA (ID 001973, all Thermo Fisher Scientific) were reverse transcribed from 10 ng RNA using Taqman miRNA Reverse Transcription kit (Thermo Fisher Scientific) according to the manufacturer’s instruction. Taqman miRNA assays were performed using the QuantStudio 3 Real-Time PCR System on 0.7 ng miRNA, together with 1 × miR-146a, miR-155, miR-223 or U6snRNA Taqman miRNA PCR probes and 1 × Taqman Universal Master Mix II, no UNG (Thermo Fisher Scientific), according to the following conditions: 95 °C for 10 min, followed by 40 cycles of 95 °C for 15 s and 60 °C for 60 s. miRNA abundance was normalized to U6snRNA expression using the delta C(t) method^82^.

### Flow cytometry

Single-cell suspensions were prepared from the dLN of *Tlr4*^+/+^ and *Tlr4*^*−/−*^ females in estrus or on d 3.5 pc, incubated with Fc receptor block (BD Biosciences), and labeled with fluorophore-conjugated monoclonal antibodies against CD4·APC-Cy7, CD25·PE-Cy7 (both BD Biosciences), NRP1·BV421 (Biolegend, San Diego, CA), then treated with Intracellular Fixation and Permeabilization Buffer Set (Thermo Fisher Scientific) and labeled with antibodies against Ki67·FITC, FOXP3·APC (both Thermo Fisher Scientific), and CTLA4·PE (BD Biosciences), and analyzed on a FACS Canto II flow cytometer with FACSDiva (BD Biosciences) and FlowJo software (BD Biosciences) as described^6^, using gating as shown in Supplemental Fig. S3. Count beads (CountBright™ Count Beads, Thermo Fisher Scientific) were used to calculate the total number of each cell type in each tissue, as described^54^. In all samples, gates were established using unlabeled, fluorescence minus one and isotype-matched negative controls.

### t-distributed stochastic neighbor embedding (tSNE) analysis

For tSNE analysis of flow cytometry data, the expression profile of CD4, FOXP3, NRP1, CD25, CTLA4, and Ki67 in singlet, viable CD4^+^ cells from dLN of mated *Tlr4*^+/+^ and *Tlr4*^*−*/*−*^ mice was utilized, using conventional gating in each sample, with dead cells, debris and off-scale (low or high) fluorescence events excluded. Samples from 25 mice in the 2 mated groups each had 20,000 viable CD4^+^ cells concatenated into a single .fcs file that was transformed in FlowJo using Barnes–Hut implementation of the tSNE algorithm^83^ with 1000 iterations, a perplexity of 25 and learning rate of 1000. X-Shift analysis^84^ in FlowJo software was used to determine unique cell clusters, using the 6 cell markers, within the viable CD4^+^ cells in the tSNE plot, using k-nearest neighbour density of 400, subsampling 100,000 cells. The MFI for each marker was assessed within each cluster, relative expression calculated and an expression heat map generated in GraphPad Prism 9.0 for Windows (GraphPad Prism Software Inc, La Jolla, CA). The proportion that each cluster contributed to the tSNE plot was obtained for each sample to compare cluster frequencies between the different treatment groups.

For tSNE analysis of endometrial cytokine mRNA expression, relative mRNA values for *Ccl2*, *Ccl3*, *Ccl19*, *Csf2*, *Csf3, Cxcl1*, *Cxcl2*, *Cxcl10*, *Il1b*, *Il6*, *Lif, Ptgs2* and *Tnf* expression were obtained from mated *Tlr4*^+/+^ and *Tlr4*^*−*/*−*^ mice as described above. Values for each of the 12 genes from each mouse were concatenated into a single .fcs file, which was transformed in FlowJo using Barnes–Hut implementation of the tSNE algorithm^83^ with 80 iterations, a perplexity of 5 and learning rate of 2. The tSNE-Y and tSNE-X values were applied to a dot plot for visualization.

### Placental histology

Serial mid-sagittal 7 μm sections were cut, placed on ten 3-aminopropyltriethoxysilane coated slides and the first slide with a full placental face was stained with Masson’s trichrome as previously described^60^. The total areas of junctional and labyrinthine zones were measured by video image analysis Images after image capture with a NanoZoomer 1.0 U (Hamamatsu, Shizuoka, Japan) and the junctional and labyrinthine zones and total placental area were assessed using FIJI Image J (Ludwig Institute for Cancer Research, New York, USA) at a zoom equivalent of a 10 × objective lens. Areas were expressed as proportion (%) of total midsagittal area comprised by junctional and labyrinthine zones.

### Immunohistology

F4/80^+^ macrophages and MHC class II^+^ DCs were detected in paraffin-embedded uterine tissue of estrus control or mated females as previously described^22^ using rat anti-mouse F4/80 (Agilent, California, USA) and rat anti-mouse MHC class II (Invitrogen, Themo Fisher Scientific). Tissue sections (5 µM) were cut on a HM 325 Rotary Microtome (Thermo Fisher Scientific), dewaxed in xylene, and rehydrated. Endogenous peroxidase activity was blocked by incubating sections in quenching solution (50% methanol, 10% H_2_O_2_) for 15 min at room temperature followed by washing in MilliQ H_2_O. Non-specific antibody binding was blocked by incubation in blocking buffer (15% normal rabbit serum (vol/vol) in PBS) for 30 min at 37 °C. Sections were washed in PBS before incubation with rat anti-F4/80, rate anti-MHC class II, or irrelevant isotype-matched control mAb (1:400 dilution in 1.5% normal rabbit serum (vol/vol) in PBS), overnight at 4 °C in a humidified chamber. Following washing in PBS, biotinylated rabbit anti-rat IgG (1:500 dilution in 1.5% normal rabbit serum in PBS; Vector Laboratories, Burlingame, CA) was added for 40 min at room temperature. Sections were washed in PBS before incubation with ABC Vectorstain Elite kit (Vector Laboratories) according to manufacturer’s instructions. Detection was performed using diaminobenzidine tetrachloride (Sigma-Aldrich), and sections were counterstained with hematoxylin (Sigma-Aldrich). Images were captured as above at a zoom equivalent of a 20 × objective lens and staining was quantified using FIJI Image J.

### Statistics

Statistical tests were carried out in GraphPad Prism 9 unless otherwise specified (GraphPad Software Suite, La Jolla, CA, USA). Normality of data was assessed using the D’Agostino and Pearson normality test where appropriate. qPCR, flow cytometry, and immunohistology data was analyzed by two-way analysis of variance (ANOVA) to detect the effects of genotype and mating, and one-way ANOVA with a post-hoc Sidak multiple comparison test to detect differences between groups. Total implantation sites, viable litter size, and resorption rate were analyzed using unpaired *t* test or non-parametric Mann–Whitney *U* test. Fetal weight, placental weight and fetal:placental weight ratio were analyzed by Mixed Model Linear Repeated-Measures ANOVA and post hoc least significant difference *t* test using SPSS software (San Diego, CA, USA), with the mother as a subject and the number of viable pups in the litter as a covariate. Differences between groups were considered significant when *p* < 0.05.

## Supplementary Information

Supplementary Figures.

## References

[1] Erlebacher A (2013). Mechanisms of T cell tolerance towards the allogeneic fetus. Nat. Rev. Immunol..

[2] Robertson SA, Care AS, Moldenhauer LM (2018). Regulatory T cells in embryo implantation and the immune response to pregnancy. J. Clin. Investig..

[3] Robertson SA (2009). Seminal fluid drives expansion of the CD4^+^CD25^+^ T regulatory cell pool and induces tolerance to paternal alloantigens in mice. Biol. Reprod..

[4] Schjenken JE, Robertson SA (2020). The female response to seminal fluid. Physiol. Rev..

[5] Schjenken JE, Glynn DJ, Sharkey DJ, Robertson SA (2015). TLR4 signaling is a major mediator of the female tract response to seminal fluid in mice. Biol. Reprod..

[6] Schjenken JE (2020). MicroRNA miR-155 is required for expansion of regulatory T cells to mediate robust pregnancy tolerance in mice. Mucosal Immunol..

[7] Robertson SA (2017). MicroRNA regulation of immune events at conception. Mol. Rerod. Dev..

[8] Robertson SA, Mau VJ, Tremellen KP, Seamark RF (1996). Role of high molecular weight seminal vesicle proteins in eliciting the uterine inflammatory response to semen in mice. J. Reprod. Fertil..

[9] Robertson SA, Allanson M, Mau VJ (1998). Molecular regulation of uterine leukocyte recruitment during early pregnancy in the mouse. Placenta.

[10] Pollard JW, Lin EY, Zhu L (1998). Complexity in uterine macrophage responses to cytokines in mice. Biol. Reprod..

[11] Moldenhauer LM (2009). Cross-presentation of male seminal fluid antigens elicits T cell activation to initiate the female immune response to pregnancy. J. Immunol..

[12] Moldenhauer LM (2019). Thymus-derived regulatory T cells exhibit Foxp3 epigenetic modification and phenotype attenuation after mating in mice. J. Immunol..

[13] Care AS (2018). Reduction in regulatory T cells in early pregnancy causes uterine artery dysfunction in mice. Hypertension.

[14] Zenclussen AC (2005). Abnormal T-cell reactivity against paternal antigens in spontaneous abortion: Adoptive transfer of pregnancy-induced CD4^+^CD25^+^ T regulatory cells prevents fetal rejection in a murine abortion model. Am. J. Pathol..

[15] Shima T (2010). Regulatory T cells are necessary for implantation and maintenance of early pregnancy but not late pregnancy in allogeneic mice. J. Reprod. Immunol..

[16] Rowe JH, Ertelt JM, Xin L, Way SS (2012). Pregnancy imprints regulatory memory that sustains anergy to fetal antigen. Nature.

[17] Guerin LR, Prins JR, Robertson SA (2009). Regulatory T-cells and immune tolerance in pregnancy: A new target for infertility treatment?. Hum. Reprod. Update.

[18] Baratelli F (2005). Prostaglandin E2 induces FOXP3 gene expression and T regulatory cell function in human CD4^+^ T cells. J. Immunol..

[19] Robertson SA, Ingman WV, O'Leary S, Sharkey DJ, Tremellen KP (2002). Transforming growth factor beta—A mediator of immune deviation in seminal plasma. J. Reprod. Immunol..

[20] Kim BJ (2015). Seminal CD38 is a pivotal regulator for fetomaternal tolerance. Proc. Natl. Acad. Sci. U.S.A..

[21] Schjenken JE, Glynn DJ, Sharkey DJ, Robertson SA (2015). TLR4 signaling is a major mediator of the female tract response to seminal fluid in mice. Biol. Reprod..

[22] Schjenken JE (2021). Sperm modulate uterine immune parameters relevant to embryo implantation and reproductive success in mice. Commun. Biol..

[23] Rutella S, Lemoli RM (2004). Regulatory T cells and tolerogenic dendritic cells: From basic biology to clinical applications. Immunol. Lett..

[24] Manicassamy S, Pulendran B (2011). Dendritic cell control of tolerogenic responses. Immunol. Rev..

[25] Hume DA (2015). The many alternative faces of macrophage activation. Front. Immunol..

[26] Imhof BA, Jemelin S, Emre Y (2017). Toll-like receptors elicit different recruitment kinetics of monocytes and neutrophils in mouse acute inflammation. Eur. J. Immunol..

[27] Bugl S (2013). Steady-state neutrophil homeostasis is dependent on TLR4/TRIF signaling. Blood.

[28] Nadkarni S (2016). Neutrophils induce proangiogenic T cells with a regulatory phenotype in pregnancy. Proc. Natl. Acad. Sci. U.S.A..

[29] Lim H (1997). Multiple female reproductive failures in cyclooxygenase 2-deficient mice. Cell.

[30] He X, Jing Z, Cheng G (2014). MicroRNAs: New regulators of Toll-like receptor signalling pathways. Biomed. Res. Int..

[31] Weiss JM (2012). Neuropilin 1 is expressed on thymus-derived natural regulatory T cells, but not mucosa-generated induced Foxp3^+^ T reg cells. J. Exp. Med..

[32] Yadav M (2012). Neuropilin-1 distinguishes natural and inducible regulatory T cells among regulatory T cell subsets in vivo. J. Exp. Med..

[33] Read S, Malmstrom V, Powrie F (2000). Cytotoxic T lymphocyte-associated antigen 4 plays an essential role in the function of CD25(+)CD4(+) regulatory cells that control intestinal inflammation. J. Exp. Med..

[34] Moldenhauer LM, Keenihan SN, Hayball JD, Robertson SA (2010). GM-CSF is an essential regulator of T cell activation competence in uterine dendritic cells during early pregnancy in mice. J. Immunol..

[35] van Mourik MS, Macklon NS, Heijnen CJ (2009). Embryonic implantation: Cytokines, adhesion molecules, and immune cells in establishing an implantation environment. J. Leuk. Biol..

[36] Dimitriadis E, White CA, Jones RL, Salamonsen LA (2005). Cytokines, chemokines and growth factors in endometrium related to implantation. Hum. Reprod. Update.

[37] Jauniaux E, Poston L, Burton GJ (2006). Placental-related diseases of pregnancy: Involvement of oxidative stress and implications in human evolution. Hum. Reprod. Update.

[38] Woods L, Perez-Garcia V, Hemberger M (2018). Regulation of placental development and its impact on fetal growth-new insights from mouse models. Front. Endocrinol. (Lausanne).

[39] Fowden AL, Forhead AJ, Coan PM, Burton GJ (2008). The placenta and intrauterine programming. J. Neuroendocrinol..

[40] Xin L (2014). Cutting edge: Committed Th1 CD4^+^ T cell differentiation blocks pregnancy-induced Foxp3 expression with antigen-specific fetal loss. J. Immunol..

[41] Moldenhauer LM, Diener KR, Hayball JD, Robertson SA (2017). An immunogenic phenotype in paternal antigen-specific CD8^+^ T cells at embryo implantation elicits later fetal loss in mice. Immunol. Cell Biol..

[42] Yudin AI (2008). Beta-defensin 22 is a major component of the mouse sperm glycocalyx. Reproduction.

[43] Shimada M (2008). Hyaluronan fragments generated by sperm-secreted hyaluronidase stimulate cytokine/chemokine production via the TLR2 and TLR4 pathway in cumulus cells of ovulated COCs, which may enhance fertilization. Development.

[44] Blois SM (2007). Dendritic cells: Key to fetal tolerance?. Biol. Reprod..

[45] Jia YP (2017). TLR2/TLR4 activation induces Tregs and suppresses intestinal inflammation caused by *Fusobacterium nucleatum* in vivo. PLoS One.

[46] Ding F (2020). Low-dose LPS induces tolerogenic Treg skewing in asthma. Front. Immunol..

[47] Bock M (2018). The posttraumatic activation of CD4^+^ T regulatory cells is modulated by TNFR2- and TLR4-dependent pathways, but not by IL-10. Cell Immunol..

[48] Du MR, Wang SC, Li DJ (2014). The integrative roles of chemokines at the maternal-fetal interface in early pregnancy. Cell Mol. Immunol..

[49] Ramhorst R (2016). Decoding the chemokine network that links leukocytes with decidual cells and the trophoblast during early implantation. Cell Adhes. Migr..

[50] von Bergwelt-Baildon MS (2006). CD25 and indoleamine 2,3-dioxygenase are up-regulated by prostaglandin E2 and expressed by tumor-associated dendritic cells in vivo: Additional mechanisms of T-cell inhibition. Blood.

[51] Schiavon V (2019). Microenvironment tailors nTreg structure and function. Proc. Natl. Acad. Sci. U.S.A..

[52] Petri B, Sanz MJ (2018). Neutrophil chemotaxis. Cell Tissue Res..

[53] Nancy P (2012). Chemokine gene silencing in decidual stromal cells limits T cell access to the maternal–fetal interface. Science.

[54] Guerin LR (2011). Seminal fluid regulates accumulation of FOXP3^+^ regulatory T cells in the preimplantation mouse uterus through expanding the FOXP3^+^ cell pool and CCL19-mediated recruitment. Biol. Reprod..

[55] Schjenken JE (2016). miRNA regulation of immune tolerance in early pregnancy. Am. J. Reprod. Immunol..

[56] Kawai T, Akira S (2007). Signaling to NF-kappaB by Toll-like receptors. Trends Mol. Med..

[57] Robertson SA, Chin PY, Schjenken JE, Thompson JG (2015). Female tract cytokines and developmental programming in embryos. Adv. Exp. Med. Biol..

[58] Robertson SA, Chin PY, Femia JG, Brown HM (2018). Embryotoxic cytokines—Potential roles in embryo loss and fetal programming. J. Reprod. Immunol..

[59] Thouas GA (2015). Soluble ligands and their receptors in human embryo development and implantation. Endocr. Rev..

[60] Sjoblom C, Roberts CT, Wikland M, Robertson SA (2005). Granulocyte-macrophage colony-stimulating factor alleviates adverse consequences of embryo culture on fetal growth trajectory and placental morphogenesis. Endocrinology.

[61] Riley JK, Heeley JM, Wyman AH, Schlichting EL, Moley KH (2004). TRAIL and KILLER are expressed and induce apoptosis in the murine preimplantation embryo. Biol. Reprod..

[62] Orr JS (2012). Toll-like receptor 4 deficiency promotes the alternative activation of adipose tissue macrophages. Diabetes.

[63] Jasper MJ (2010). Macrophage-derived LIF and IL1B regulate alpha(1,2)fucosyltransferase 2 (Fut2) expression in mouse uterine epithelial cells during early pregnancy. Biol. Reprod..

[64] Smith SD, Dunk CE, Aplin JD, Harris LK, Jones RL (2009). Evidence for immune cell involvement in decidual spiral arteriole remodeling in early human pregnancy. Am. J. Pathol..

[65] Zhang YH, He M, Wang Y, Liao AH (2017). Modulators of the balance between M1 and M2 macrophages during pregnancy. Front. Immunol..

[66] Zhou S (2018). SjHSP60 induces CD4(+) CD25(+) Foxp3(+) Tregs via TLR4-Mal-drived production of TGF-beta in macrophages. Immunol. Cell Biol..

[67] Perdijk O, van Neerven RJJ, Meijer B, Savelkoul HFJ, Brugman S (2018). Induction of human tolerogenic dendritic cells by 3′-sialyllactose via TLR4 is explained by LPS contamination. Glycobiology.

[68] Steinman RM, Hawiger D, Nussenzweig MC (2003). Tolerogenic dendritic cells. Annu. Rev. Immunol..

[69] Rutella S, Zavala F, Danese S, Kared H, Leone G (2005). Granulocyte colony-stimulating factor: A novel mediator of T cell tolerance. J. Immunol..

[70] Abrahams VM, Mor G (2005). Toll-like receptors and their role in the trophoblast. Placenta.

[71] Chin PY (2019). Toll-like receptor-4 antagonist (+)-naloxone confers sexually dimorphic protection from inflammation-induced fetal programming in mice. Endocrinology.

[72] Wahid HH (2015). Toll-like receptor 4 is an essential upstream regulator of on-time parturition and perinatal viability in mice. Endocrinology.

[73] Robertson SA, Care AS, Skinner RJ (2007). Interleukin 10 regulates inflammatory cytokine synthesis to protect against lipopolysaccharide-induced abortion and fetal growth restriction in mice. Biol. Reprod..

[74] Friebe A (2011). Neutralization of LPS or blockage of TLR4 signaling prevents stress-triggered fetal loss in murine pregnancy. J. Mol. Med..

[75] Lin Y, Xie M, Chen Y, Di J, Zeng Y (2006). Preterm delivery induced by LPS in syngeneically impregnated BALB/c and NOD/SCID mice. J. Reprod. Immunol..

[76] Chin PY (2016). Novel Toll-like receptor-4 antagonist (+)-naloxone protects mice from inflammation-induced preterm birth. Sci. Rep..

[77] Wahid HH (2020). Toll-like receptor-4 antagonist (+)-naltrexone protects against carbamyl-platelet activating factor (Cpaf)-induced preterm labor in mice. Am. J. Pathol..

[78] Barboza R (2017). TLR4-mediated placental pathology and pregnancy outcome in experimental malaria. Sci. Rep..

[79] Carbillon L (2016). Fetal/placental weight ratio in a mouse model of maternal diet-induced obesity. Proc. Natl. Acad. Sci. U.S.A..

[80] Thaete LG (2013). Impact of toll-like receptor 4 deficiency on the response to uterine ischemia/reperfusion in mice. Reproduction.

[81] Byers SL, Wiles MV, Dunn SL, Taft RA (2012). Mouse estrous cycle identification tool and images. PLoS One.

[82] Livak KJ, Schmittgen TD (2001). Analysis of relative gene expression data using real-time quantitative PCR and the 2(−Delta Delta C(T)) method. Methods.

[83] van der Maaten L, Hinton G (2008). Visualizing data using t-SNE. J. Mach. Learn. Res..

[84] Samusik N, Good Z, Spitzer MH, Davis KL, Nolan GP (2016). Automated mapping of phenotype space with single-cell data. Nat. Methods.

