# Supplementary material for: Toll-like receptor-4 null mutation causes fetal loss and fetal growth restriction associated with impaired maternal immune tolerance in mice
Source: Sci Rep. 2021 Aug 16;11:16569. doi: 10.1038/s41598-021-95213-1 (PMC8368181; doi:10.1038/s41598-021-95213-1)
Supplement: Supplementary file 1 — Supplementary Figures. [file 41598_2021_95213_MOESM1_ESM.pdf]

Supplementary Material

Toll-like receptor-4 null mutation causes fetal loss and fetal growth restriction associated with impaired maternal immune tolerance in mice

Hon Yeung Chan[1#], Lachlan M. Moldenhauer[1#], Holly M. Groome[1], John S. Schjenken[1,2], and Sarah A. Robertson[1*]
[#]These authors contributed equally

[1]Robinson Research Institute and Adelaide Medical School, University of Adelaide, Adelaide, SA 5005, Australia.
[2]The Priority Research Centre for Reproductive Science and School of Environmental and Life Sciences, University of Newcastle, NSW 2308, Australia.

**Correspondence and requests for materials should be addressed to:*
Sarah A. Robertson, PhD
Robinson Research Institute and Adelaide Medical School
University of Adelaide, Adelaide, SA 5005 AUSTRALIA.
Phone: +61 8 8313 4094; Fax: +61 8 8313 4099
Email: sarah.robertson@adelaide.edu.au

This pdf file includes:

Supplementary Figures S1-S5

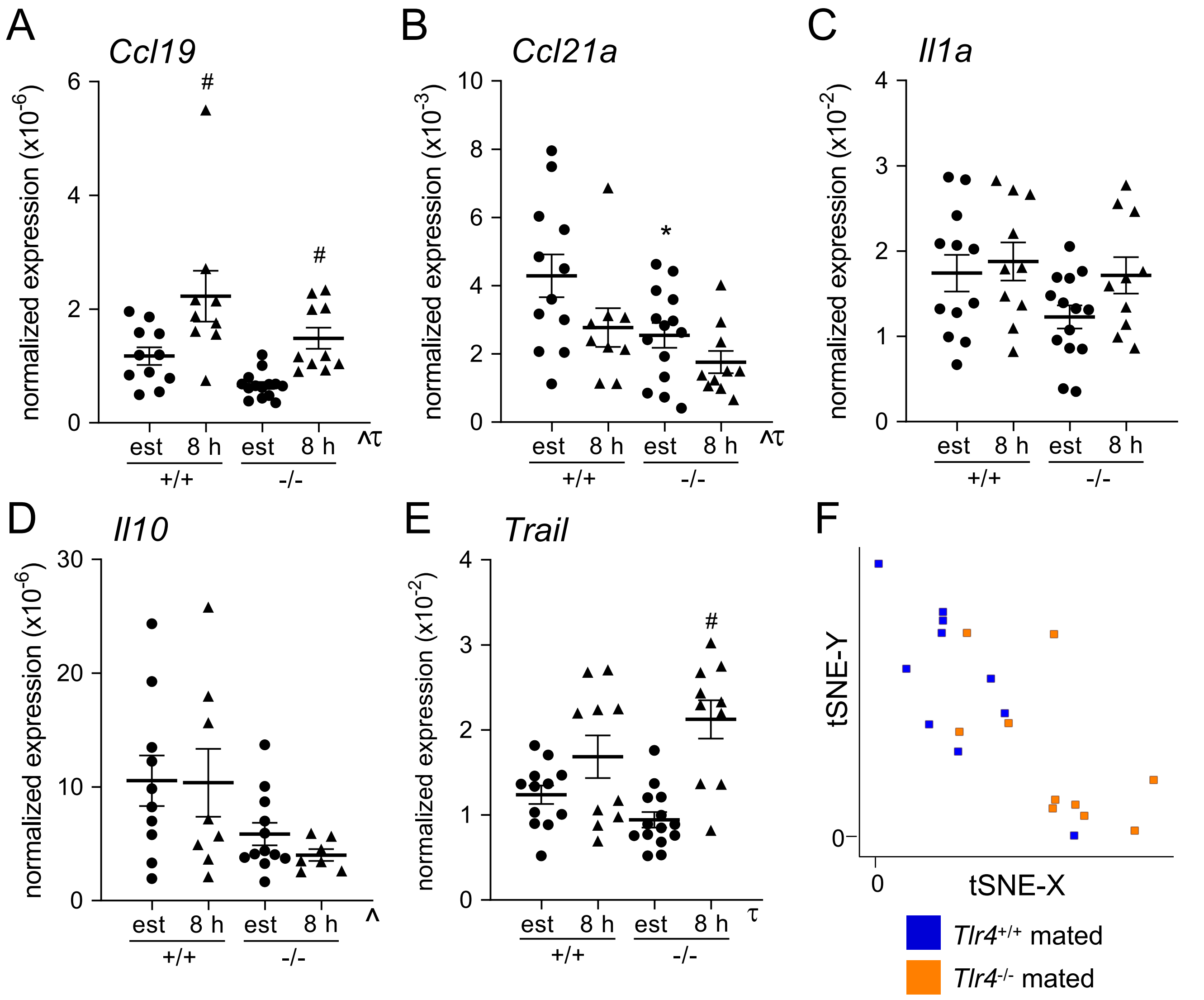

Figure S1. Effect of maternal TLR4 deficiency on mating-induced expression of cytokine and immune regulatory genes in the uterine endometrium. mRNA recovered from endometrial tissue of *Tlr4*$^{+/+}$ (+/+) and *Tlr4*$^{-/-}$ (-/-) mice at estrus, or at 8 h after mating with B6 males, was reverse transcribed into cDNA and analyzed by qPCR to quantify cytokine gene expression. Expression was normalized to *Actb* using the delta C(t) method. (**A**) *Ccl19*, (**B**) *Ccl21a*, (**C**) *Il1a*, (**D**) *Il10*, and (**E**) *Trail*. Symbols depict individual mice (n = 10-14/group) with mean ± SEM also shown. Effects of mating and genotype were assessed by one-way ANOVA with post-hoc Sidak's multiple comparison test (*$p < 0.05$ versus same time point in *Tlr4*$^{+/+}$ females, $^{\#}p < 0.05$ versus estrus within genotype). The overall effect of genotype and mating was assessed by two-way ANOVA (^$p < 0.05$ difference attributable to genotype; $^{\tau}p < 0.05$ difference attributable to mating). For tSNE analysis of qPCR data (F), values for *Ccl2*, *Ccl3*, *Ccl19*, *Csf2*, *Csf3, Cxcl1*, *Cxcl2*, *Cxcl10*, *Il1b*, *Il6*, *Lif, Tnf* and *Ptgs2* expression for each d 3.5 pc mated *Tlr4*$^{+/+}$ and *Tlr4*$^{-/-}$ mouse were concatenated into a single .fcs file and transformed by the tSNE algorithm (FlowJo software version 10.6.1, BD Biosciences, Ashland, OR), and displayed on a dot plot for visualization, where each symbol represents an individual mouse (n = 10/group).

FIGURE S1

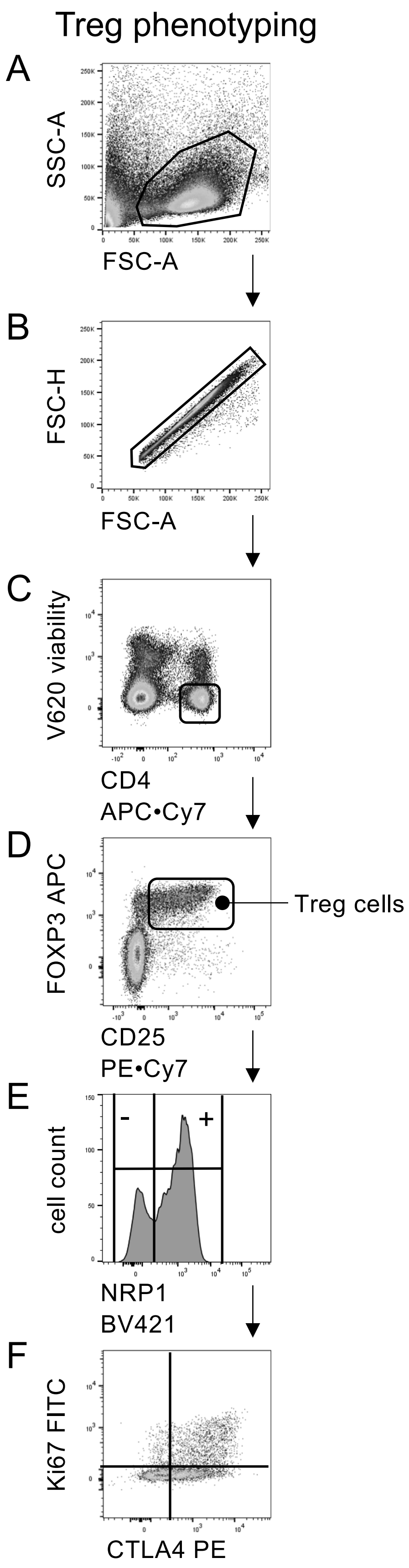

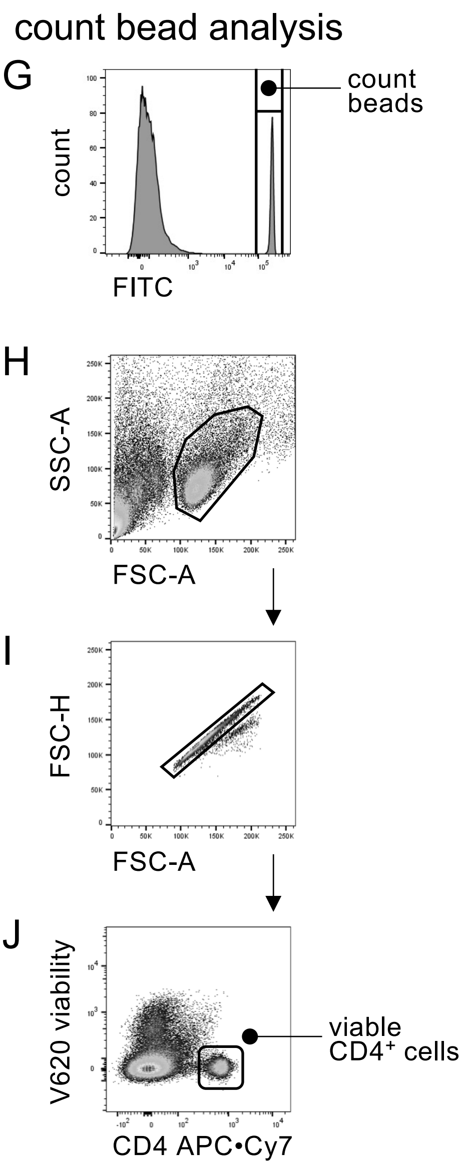

Figure S2. Gating strategy for flow cytometry. T cell phenotypes were assessed by flow cytometry with gates established on scatter plots to include lymphocytes and exclude debris and doublets (**A,B**). Viable CD4$^+$ T cells were identified (**C**) and applied to a FOXP3 vs. CD25 plot to define FOXP3$^+$CD25$^+$ Treg cells (**D**). NRP1 expression in the Treg cell population was assessed (**E**), then total CD4$^+$FOXP3$^+$CD25$^+$ Treg, CD4$^+$FOXP3$^+$NRP1$^+$ tTreg and CD4$^+$FOXP3$^+$NRP1$^-$ pTreg cells analysed for Ki67 and CTLA4 expression (**F**). Total cell counts were achieved using count beads, with all events plotted on a histogram to identify and enumerate count beads based on their very high fluorescence (**G**). Scatter plots (**H, I**) were used to detect lymphocytes and exclude debris and doublets, with cells applied to a viability vs. CD4 plot to identify and quantify viability of CD4$^+$ cells (**J**).

FIGURE S2

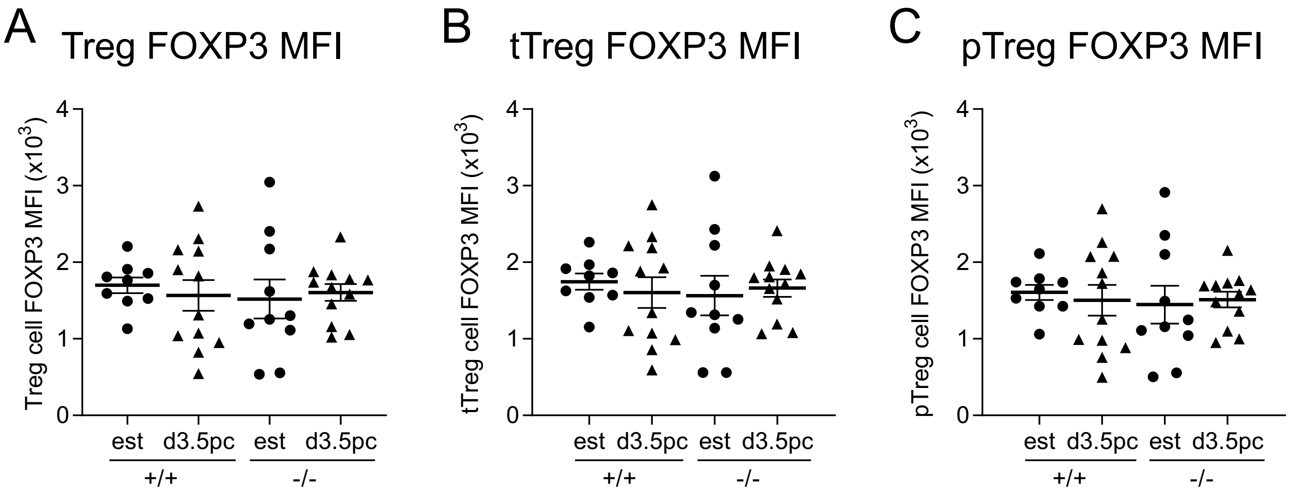

FIGURE S3. Effect of maternal TLR4 deficiency on FOXP3 expression in Treg, tTreg, and pTreg cells in the uterine dLN after mating. Cells harvested from the dLN of *Tlr4*$^{+/+}$ (+/+) and *Tlr4*$^{-/-}$ (-/-) mice at estrus, or on d 3.5 pc after mating with B6 males, were analyzed by flow cytometry to assess phenotype of Treg cells (defined as CD4^{+}CD25^{+}FOXP3^{+}), tTreg (NRP1^{+} Treg), and pTreg (NRP1^{-} Treg) cells (see Figure 2). FOXP3 MFI was assessed within the total Treg (**A**), tTreg (**B**) and pTreg (**C**) populations. Symbols depict individual mice (n = 10-17/group) and data are shown as mean ± SEM. Differences between groups were assessed by one-way ANOVA with Sidak t-test. No effect of mating or genotype on FOXP3 MFI was seen.

FIGURE S3

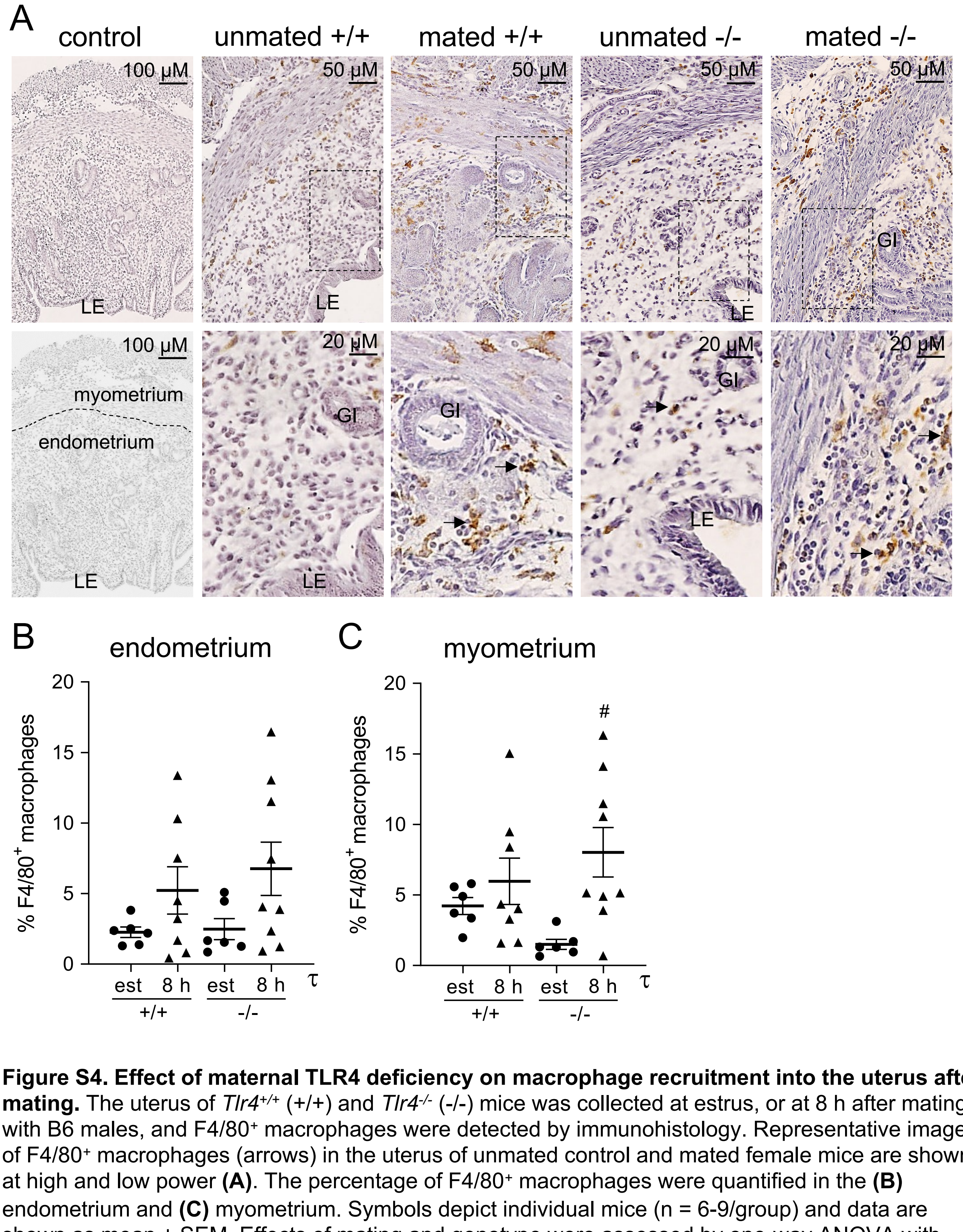

Figure S4. Effect of maternal TLR4 deficiency on macrophage recruitment into the uterus after mating. The uterus of *Tlr4*$^{+/+}$ (+/+) and *Tlr4*$^{-/-}$ (-/-) mice was collected at estrus, or at 8 h after mating with B6 males, and F4/80^{+} macrophages were detected by immunohistology. Representative images of F4/80^{+} macrophages (arrows) in the uterus of unmated control and mated female mice are shown, at high and low power **(A)**. The percentage of F4/80^{+} macrophages were quantified in the **(B)** endometrium and **(C)** myometrium. Symbols depict individual mice (n = 6-9/group) and data are shown as mean ± SEM. Effects of mating and genotype were assessed by one-way ANOVA with post-hoc Sidak's multiple comparison test ($^{\#}p < 0.05$ versus estrus within genotype). The overall effect of genotype and mating was assessed by two-way ANOVA ($^{\tau}p < 0.05$ difference attributable to mating). Isotype-matched negative control, with endometrium, myometrium, and luminal epithelium compartments, are also shown. LE = luminal epithelium; GI = epithelial glands.

FIGURE S4

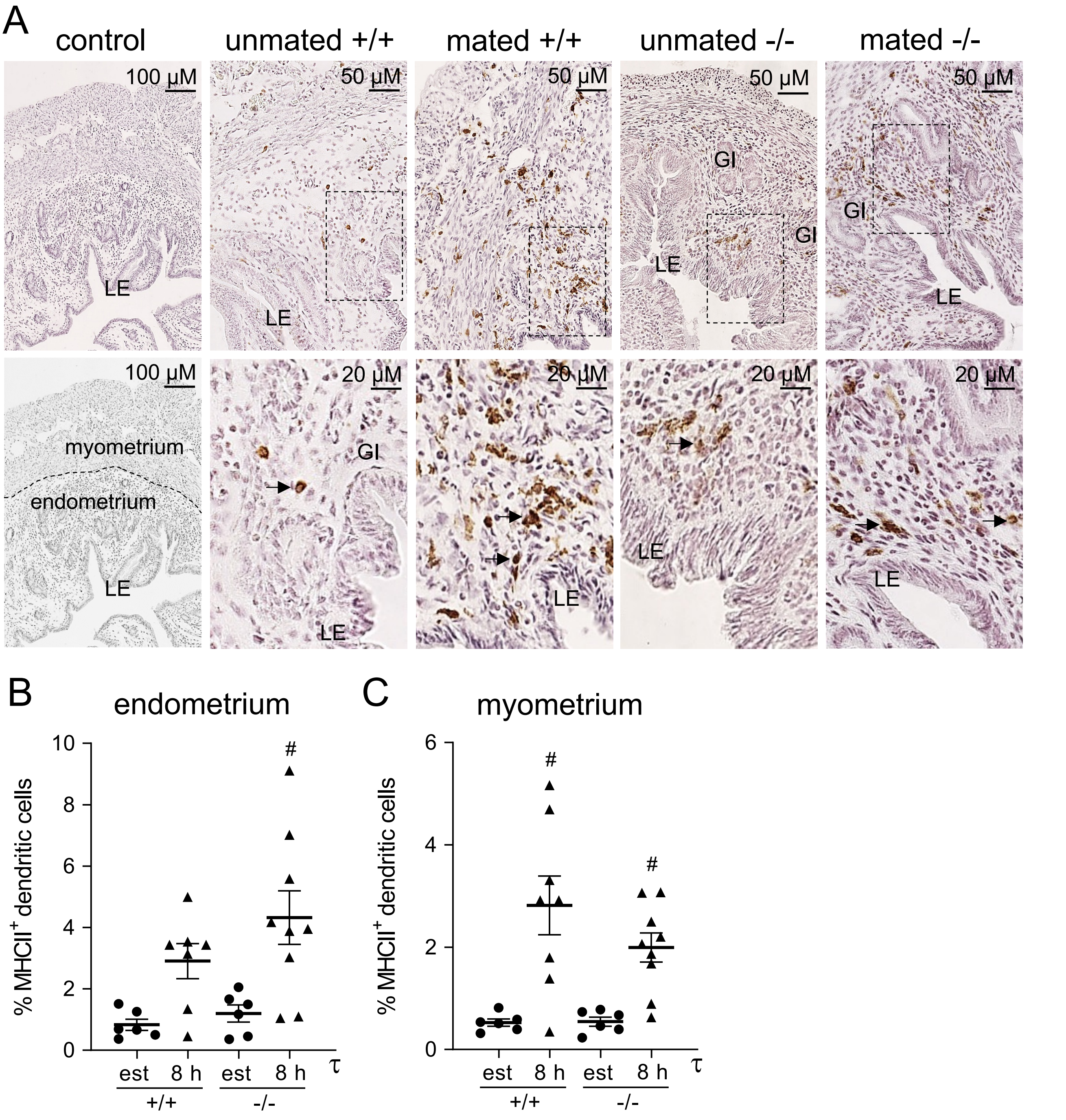

Figure S5. Effect of maternal TLR4 deficiency on dendritic cell recruitment into the uterus after mating. The uterus of $Tlr4^{+/+}$ (+/+) and $Tlr4^{-/-}$ (-/-) mice was collected at estrus, or at 8 h after mating with B6 males, and MHC class II^{+} dendritic cells were detected by immunohistology. Representative images of MHC class II^{+} dendritic cells (arrows) in the uterus of unmated control and mated female mice are shown, at high and low power **(A)**. The percentage of MHC class II^{+} dendritic cells were quantified in the **(B)** endometrium and **(C)** myometrium. Symbols depict individual mice (n = 6-9/group) and data are shown as mean ± SEM. Effects of mating and genotype were assessed by one-way ANOVA with post-hoc Sidak's multiple comparison test ($^{\#}p < 0.05$ versus estrus within genotype). The overall effect of genotype and mating was assessed by two-way ANOVA ($^{\tau}p < 0.05$ difference attributable to mating). Isotype-matched negative control, with endometrium, myometrium, and luminal epithelium compartments, are also shown. LE = luminal epithelium; GI = epithelial glands.

FIGURE S5
